# Supplementary material for: Amazon forests capture high levels of atmospheric mercury pollution from artisanal gold mining
Source: Nat Commun. 2022 Jan 28;13:559. doi: 10.1038/s41467-022-27997-3 (PMC8799693; doi:10.1038/s41467-022-27997-3)
Supplement: Supplementary file 1 — Supplementary Information [file 41467_2022_27997_MOESM1_ESM.pdf]

Supplementary Information

Figures

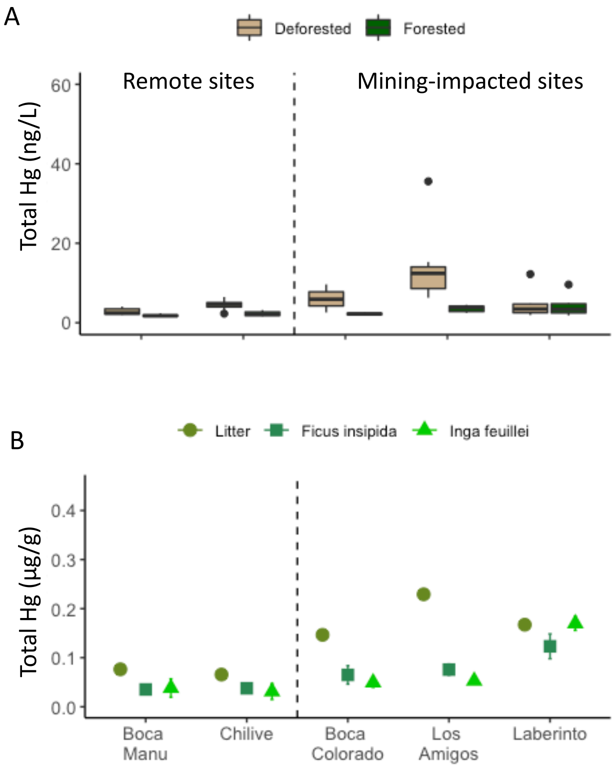

Supplementary Figure 1: A) Concentrations of total mercury in precipitation collected in forested (throughfall; brown boxplots) and deforested (bulk precipitation; green boxplots) areas during the 2018 wet season at five sites across Madre de Dios, Peru. For all boxplots, the line represents the median value, the box shows Q1 and Q3, and the whiskers denote 1.5 times the interquartile range (n=8 independent samples for each forested site, n=6 independent samples for each deforested site). B) Concentrations of total mercury in leaves collected during the 2018 wet season as bulk litter on the ground (olive green circle), from the canopy of *ficus insipida* (dark green square), and from the canopy of *inga feuillei* (light green triangle). Values are shown as mean and standard deviation (n=3 independent samples for live leaves for each site, n=1 independent sample for litter). The dashed line represents the demarcation between the two remote sites (on the left) and the three mining-impacted sites (on the right).

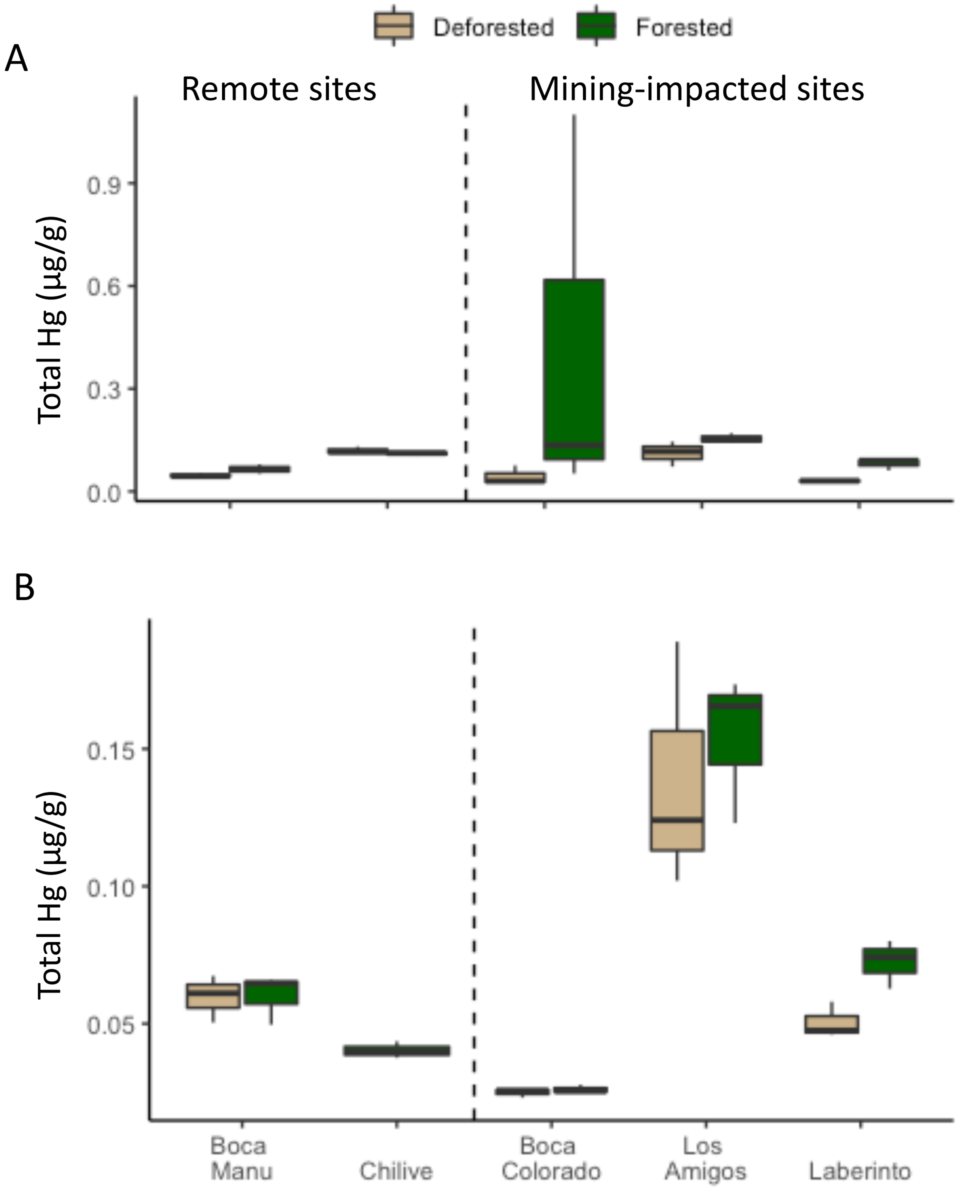

Supplementary Figure 2: Concentrations of total mercury in surficial soils (0-5 cm) collected in forested (green boxplots) and deforested (brown boxplots) areas during A) the 2018 wet season and B) 2019 dry season at five sites across Madre de Dios, Peru. The dashed line represents the demarcation between the two remote sites (on the left) and the three mining-impacted sites (on the right). For all boxplots, the line represents the median value, the box shows Q1 and Q3, and the whiskers denote 1.5 times the interquartile range (n=3 independent samples for each site in each season).

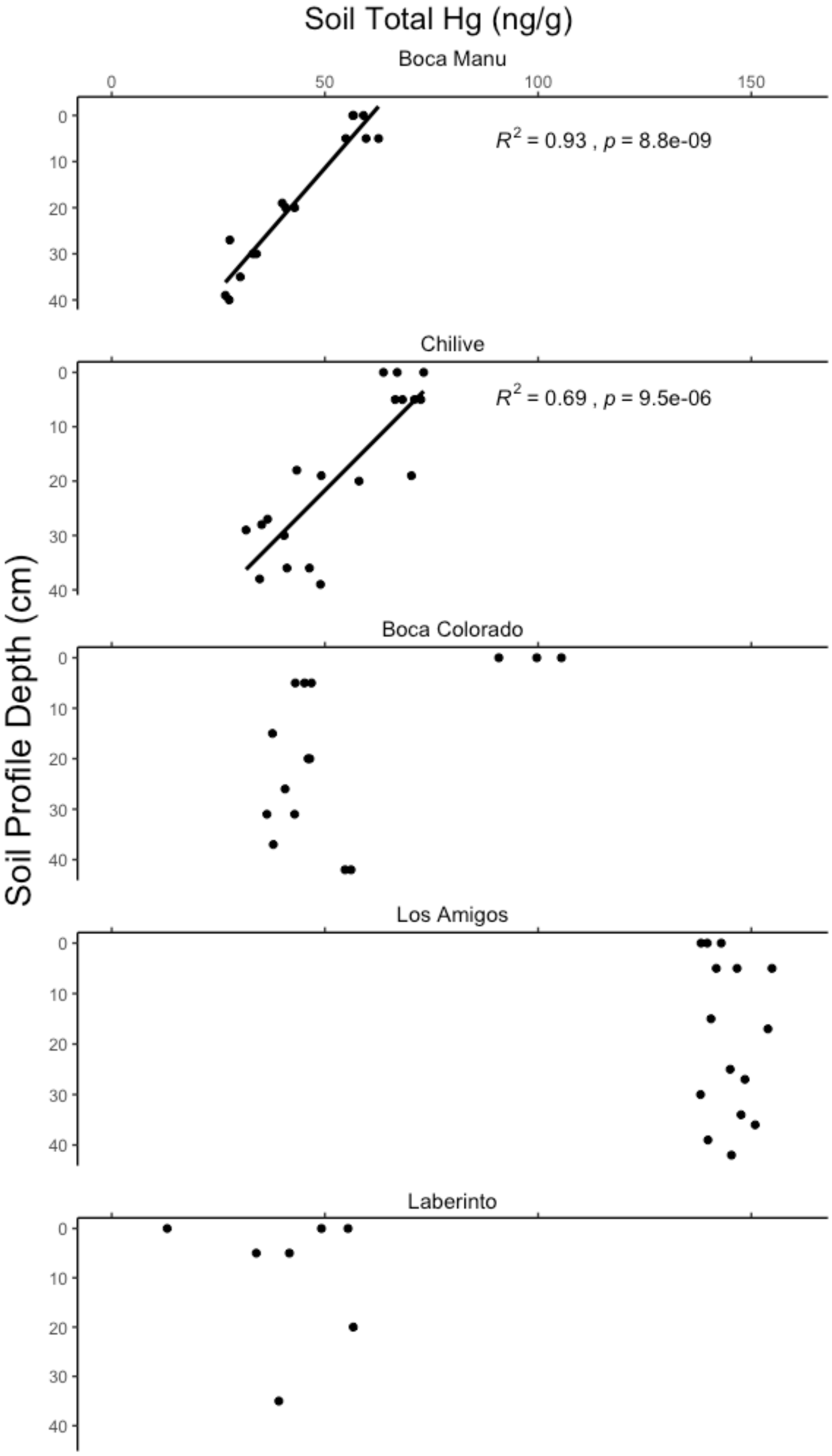

Supplementary Figure 3: Concentrations of total mercury with depth in soil profiles at the five sampling sites in Madre de Dios, Peru for the 2018 dry season. Statistical data represent ordinary least square regressions.

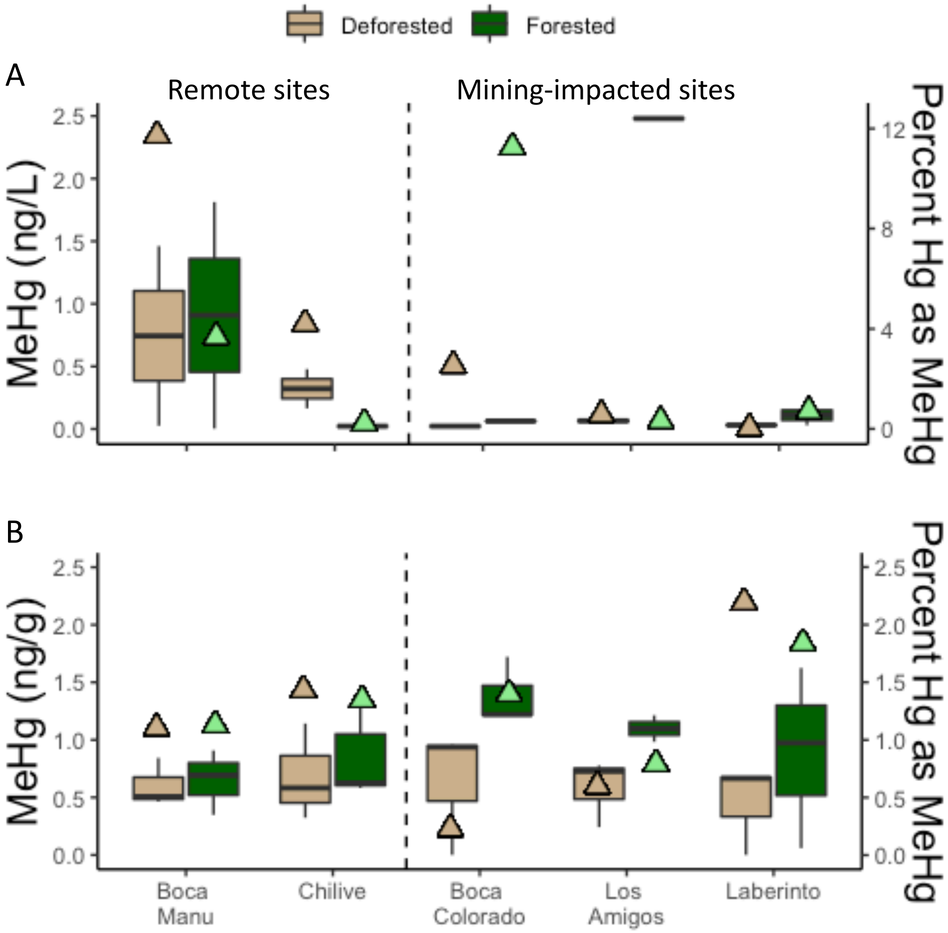

Supplementary Figure 4: Concentrations of methylmercury (MeHg; boxplots) and average percent of mercury present as methylmercury (triangles) in A) precipitation and B) surficial soil (0-5 cm) gathered in forested (green color) and deforested (brown color) areas during the 2018 dry season at five sites across Madre de Dios, Peru. For all boxplots, the line represents the median value, the box shows Q1 and Q3, and the whiskers denote 1.5 times the interquartile range. The dashed line represents the demarcation between the two remote sites (on the left) and the three mining-impacted sites (on the right).

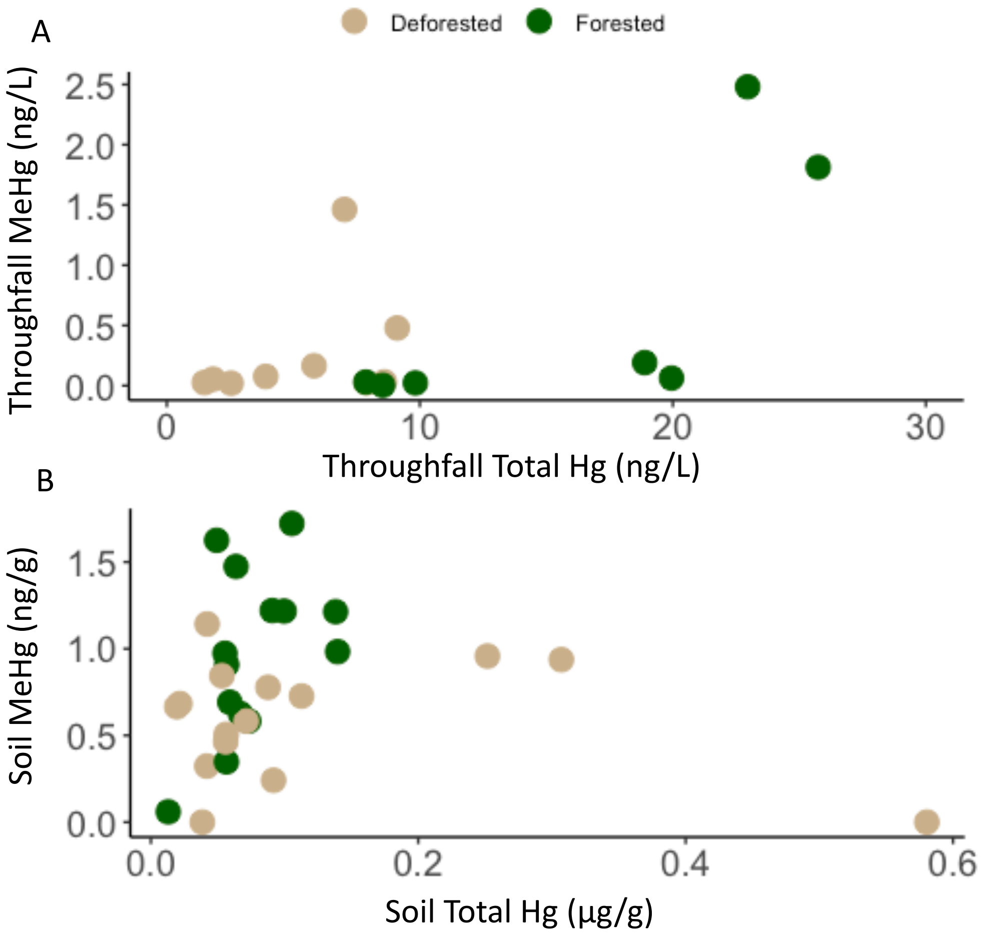

Supplementary Figure 5: Relationship between methylmercury (MeHg) and total mercury concentrations in forested (green circles) and deforested (brown circles) areas for the 2018 dry season at all five sites in Madre de Dios, Peru in A) throughfall and B) soil.

Tables

Supplementary Table 1: Total mercury flux and pool values for Los Amigos Conservation Concession in forested and deforested areas. For each calculation, the summary statistic (average, minimum, or maximum) was calculated using data for each of the dry season (2018 and 2019) and wet season (2018), and then these two values were averaged to determine an overall average, average minimum, or average maximum value that reflects data from all seasons and years. The average value in the table corresponds to the values reported in the text.

	Throughfall Flux (μg m^{-2} yr^{-1})	Litterfall Flux (μg m^{-2} yr^{-1})	Total Flux in Forested Areas (μg m^{-2} yr^{-1})	Surficial (0-5 cm) Soil Pool in Forested Areas (μg m^{-2})	Rainfall Flux/Total Flux in Deforested Areas (μg m^{-2} yr^{-1})	Surficial (0-5 cm) Soil Pool in Deforested Areas (μg m^{-2})
Average	71	66	137	9.1	8.6	7.1
Minimum	38	63	101	7.4	5.2	5.7
Maximum	138	72	210	10.7	13.8	9.1

Supplementary Table 2: Geographic coordinates for five forested sampling sites in Madre de Dios.

**Site**	**Latitude**	**Longitude**
Boca Manu	-12.26758	-70.88566
Chilive	-12.48094	-70.58096
Los Amigos	-12.56632	-70.10175
Colorado	-12.60734	-70.34124
Laberinto	-12.72128	-69.58601

Supplementary Table 3: Geographic coordinates for Los Amigos plots and leaf area index. Plot numbers 1 and 2 correspond to the sites sampled as part of the regional Madre de Dios sampling (Supplementary Table 2).

**Plot Number**	**Type**	**Latitude**	**Longitude**	**Leaf Area Index (LAI; m^2 leaf area m^{-2} ground area)**
1	deforested	-12.56908	-70.09974	2.3235
2	forested	-12.56632	-70.10175	15.7752
3	forested	-12.56346	-70.10456	11.3248
4	forested	-12.56259	-70.10592	9.51443
5	forested	-12.56251	-70.10094	14.1093
6	forested	-12.57465	-70.09599	7.38483
7	forested	-12.5806	-70.09288	11.1023
8	forested	-12.58025	-70.09205	12.2801

Supplementary Table 4: Gaseous elemental mercury (GEM) concentration collected from five sites across Madre de Dios during the 2018 dry season (July-August, n=1 per site) and wet season (December-January, n=2 per site).

**Site**	**Season**	**Year**	**GEM (ng m^{-3})**
Boca Manu	dry season	2018	0.92
Chilive	dry season	2018	0.8
Colorado	dry season	2018	10.87
Los Amigos	dry season	2018	2.63
Laberinto	dry season	2018	9.55
Boca Manu	wet season	2018	0.90
Boca Manu	wet season	2018	0.82
Chilive	wet season	2018	0.87
Chilive	wet season	2018	0.95
Colorado	wet season	2018	2.26
Colorado	wet season	2018	2.62
Los Amigos	wet season	2018	1.66
Los Amigos	wet season	2018	1.56
Laberinto	wet season	2018	9.70
Laberinto	wet season	2018	9.57

Supplementary Table 5: Total mercury concentration in bulk litter and litterbaskets collected from the Los Amigos plots during the 2019 dry season (July-August).

**Site Number**	**Type**	**Total mercury concentration (μg/g)**
2	litter basket	0.17
2	bulk litter	0.24
3	litter basket	0.22
3	litter basket	0.18
3	bulk litter	0.26
4	litter basket	0.28
4	litter basket	0.15
4	bulk litter	0.13
5	litter basket	0.15
5	litter basket	0.24
5	bulk litter	0.22
6	litter basket	0.15
6	litter basket	0.15
6	bulk litter	0.22
7	litter basket	0.15
7	litter basket	0.20
7	bulk litter	0.19
8	litter basket	0.17
8	bulk litter	0.16

Título: Los bosques amazónicos capturan altos niveles de contaminación atmosférica por mercurio de la minería de oro artesanal

Autores: Jacqueline R Gerson,[1,2*] Natalie Szponar,[3] Angelica Almeyda Zambrano,[4] Bridget Bergquist,[3] Eben Broadbent,[4] Charles T Driscoll,[5] Gideon Erkenswick,[6,7] David C Evers,[9] Luis E Fernandez,[10,11,12] Heileen Hsu-Kim,[13] Giancarlo Inga,[8] Kelsey N Lansdale,[14] Melissa J Marchese,[1,2] Ari Martinez,[15] Caroline Moore,[8] William K Pan,[2,16] Raúl Pérez Purizaca,[17] Victor Sánchez,[18] Miles Silman,[10,11,12] Emily A Ury,[1] Claudia Vega,[10,11,12] Mrinalini Watsa,[7,8] Emily S Bernhardt[1]

***** Autor correspondiente; FFSC 3304, Duke University, Durham NC 27708, 732-710-1844, jgerson1@gmail.com

Afiliaciones:

[1] Department of Biology, Duke University, Durham NC 27708

[2] Duke Global Health Institute, Duke University, Durham NC 27708

[3] Department of Earth Sciences, University of Toronto, Toronto Canada M5S 3B1

[4] School of Forest Resources and Conservation, University of Florida, Gainesville FL 32611

[5] Department of Civil and Environmental Engineering, Syracuse University, Syracuse NY 13244

[6] Department of Molecular Microbiology, Washington University School of Medicine, St. Louis MO 63110

[7] Field Projects International, Escondido CA 92029

[8] San Diego Zoo Wildlife Alliance, San Diego CA 92101

[9] Biodiversity Research Institute, Portland ME 04103

[10] Centro de Innovación Científica Amazónica (CINCIA), Puerto Maldonado Perú

[11] Center for Energy, Environment, and Sustainability (CEES), Wake Forest University, Winston-Salem NC 27109

[12] Department of Biology, Wake Forest University, Winston-Salem NC 27109

[13] Department of Civil and Environmental Engineering, Duke University, Durham NC 27708

[14] Environmental Science Program, Duke University, Durham NC 27708

[15] Department of Biological Sciences, California State University, Long Beach CA 90840

[16] Nicholas School of the Environment, Duke University, Durham NC 27708
[17]Universidad Nacional de Piura, Piura Perú
[18] Instituto de Investigación en Ecología y Conservación (IIECOO), La Libertad Perú

Información de contacto:

Jacqueline R Gerson, 732-710-1844, jgerson1@gmail.com
Natalie Szponar, 647-613-2546, natalie.szponar@mail.utoronto.ca
Angelica Almeyda Zambrano, 650-204-1052, aalmeyda@ufl.edu
Bridget Bergquist, 416-978-4851, bergquist@es.utoronto.edu
Eben Broadbent, 650-204-1051, eben@ufl.edu
Charles T Driscoll, 315-443-3434, ctdrisco@syr.edu
Gideon Erkenswick, 314-649-8636, Gideon.e@wustl.edu
David Evers, 207-839-7600 x 221, david.evers@briloon.org
Luis E Fernandez, 734-678-4329, fernanle@wfu.edu
Heileen Hsu-Kim, 919-660-5109, hsukim@duke.edu
Giancarlo Inga, +51 982-417-245, giancarloingadiaz@gmail.com
Kelsey Lansdale, 210-452-1366, kelseylansdale@gmail.com
Melissa J Marchese, 732-996-6909, melissa.marchese@duke.edu
Ari Martinez, 352-275-6169, arimartinez043@gmail.com
Caroline Moore, 831-239-9942, camoore@sandiegozoo.org
William Pan, 919-684-4108, william.pan@duke.edu
Raúl Pérez Purizaca, +51 968-732-706, r.perezpurizaca@gmail.com
Victor Sánchez, +51 969-950-333, victor.sanzca@gmail.com
Miles Silman, 336-407-2139, silmanmr@wfu.edu
Emily A Ury, 413-717-1222, ury.emily@gmail.com
Claudia Vega, +51 993-714-173, vegacm@wfu.edu
Mrinalini Watsa, 224-795-3228, merkenswickwatsa@sandiegozoo.org
Emily S Bernhardt, 919-660-7318, emily.bernhardt@duke.edu

Resumen:
Las emisiones de mercurio de la minería de oro artesanal y en pequeña escala en todo el Sur Global superan a la combustión de carbón como la mayor fuente mundial de mercurio. Examinamos la deposición y almacenamiento de mercurio en un área de la Amazonía peruana fuertemente impactada por la extracción de oro artesanal. Los bosques prístinos en la Amazonía peruana cerca de la minería de oro reciben aportes extremadamente altos de mercurio y experimentan niveles elevados de mercurio total y metilmercurio en la atmósfera, el follaje del dosel y el suelo. En este artículo mostramos por primera vez que un dosel de bosque prístino cerca de la minería de oro artesanal intercepta grandes cantidades de partículas de mercurio y mercurio gaseoso, a una tasa proporcional con el área foliar total. Documentamos la acumulación sustancial de mercurio en suelos, biomasa y pájaros cantores residentes en algunas de las áreas más protegidas y biodiversas de la Amazonía, lo que plantea importantes preguntas sobre cómo la contaminación por mercurio puede limitar los esfuerzos de conservación actuales y futuros en estos ecosistemas tropicales.

Introducción

La minería de oro artesanal y en pequeña escala (MAPE) constituye un desafío creciente para los ecosistemas forestales tropicales. Esta forma de extracción de oro ocurre en más de 70 países, es con frecuencia una actividad informal o ilegal y representa aproximadamente el 20% de la producción mundial de oro. [1] Si bien la MAPE es una importante fuente de sustento para las comunidades locales, también resulta en una deforestación generalizada, [2,3] una conversión extensa de bosques en lagunas, [4] una alta carga de sedimentos en los ríos cercanos, [5,6] y la mayor fuente mundial de emisiones de mercurio atmosférico y descargas de Hg en aguas dulces. [7]

Muchos sitios de MAPE intensivos se encuentran dentro de regiones megadiversas y conducen a una disminución de la biodiversidad, [8] una pérdida de especies sensibles, [9] y una alta exposición al Hg tanto en personas [10–12] como en depredadores principales. [13,14] A nivel mundial, se estima que entre 675 y 1000 toneladas de Hg al año se volatilizan y emiten a la atmósfera a partir de las operaciones de MAPE. [7] Este uso de enormes cantidades de Hg en la MAPE ha desplazado la mayor fuente de emisión de Hg atmosférico del Norte Global al Sur Global, con consecuencias en los patrones de destino, transporte y exposición del Hg. Sin embargo, poco se sabe sobre el destino de estas emisiones de Hg atmosférico y los patrones de deposición y acumulación en las regiones afectadas por la MAPE.

El Convenio de Minamata sobre el mercurio entró en vigor en 2017 con el artículo 7 específicamente dirigido a las emisiones y descargas de Hg por la MAPE. En la MAPE, se agrega Hg elemental líquido a los sedimentos o minerales para aislar el oro. Esta amalgama se calienta posteriormente, lo que concentra el oro y libera Hg elemental gaseoso (MEG; Hg^0) a la atmósfera. La quema de amalgama a menudo ocurre sin una retorta u otro dispositivo de captura de Hg, a pesar de los esfuerzos de grupos como la Asociación Mundial sobre el Mercurio del Programa de las Naciones Unidas para el Medio Ambiente (PNUMA), la Organización de las Naciones Unidas para el Desarrollo Industrial (ONUDI) y organizaciones no gubernamentales para motivar a los mineros a mitigar las emisiones de Hg. Al momento de escribir este artículo en 2021, 132 países, incluido el Perú, han firmado el Convenio de Minamata y han comenzado a desarrollar Planes de Acción Nacionales para abordar específicamente las reducciones de Hg asociadas a la MAPE. Diferentes académicos han solicitado que estos Planes de Acción Nacionales sean inclusivos, continuos y holísticos, considerando tanto los factores socioeconómicos como los daños ambientales.[15–18] Los planes actuales para abordar las

consecuencias del Hg en el medio ambiente se centran en los riesgos de Hg asociados a la MAPE cerca de ecosistemas acuáticos, involucrando a los mineros y a quienes viven cerca de la quema de amalgama y a comunidades que consumen grandes cantidades de peces de niveles tróficos superiores. La exposición ocupacional al Hg a través de la inhalación de vapores de Hg provenientes de la quema de amalgama, la exposición al Hg en la dieta a través del consumo de pescado y la bioacumulación de Hg en la red alimentaria acuática han sido el foco de la mayoría de los estudios científicos relacionados con la MAPE, incluidos los primeros estudios en la Amazonía (véase, por ejemplo, Lodenius y Malm [15]).

Los ecosistemas terrestres también están en riesgo de exposición al Hg de la MAPE. Hay tres formas principales por las que el Hg atmosférico liberado por la MAPE como MEG puede regresar a la superficie terrestre [16] (Figura 1): el MEG puede absorberse en partículas atmosféricas, que luego son interceptadas por las superficies; las plantas pueden absorber MEG directamente e incorporarlo a sus tejidos; finalmente, el MEG se puede oxidar a especies de Hg(II) que son removidas por deposición seca, absorbidas por partículas atmosféricas o incorporadas al agua de lluvia. Estas formas de deposición suministran Hg a los suelos a través de la trascolación (es decir, la precipitación que pasa a través del dosel), la hojarasca y la lluvia, respectivamente. La deposición húmeda se puede determinar por el flujo de Hg recolectado en los claros. La deposición seca se puede determinar mediante la suma del flujo de Hg en la hojarasca y la trascolación menos el flujo de Hg en la precipitación. [17] Numerosos estudios documentan el enriquecimiento de Hg en los ecosistemas terrestres y acuáticos adyacentes a la actividad de la MAPE (por ejemplo, véase la tabla resumen en Gerson et al. [18]), que probablemente sea resultado tanto de las deposiciones atmosféricas de Hg como de las emisiones directas de Hg. No obstante, aunque la deposición mejorada de Hg cerca de la MAPE

probablemente se deba a la quema de amalgamas de Hg-Au, no está claro cómo se transporta este Hg en el paisaje regional y la importancia relativa de las diferentes formas de deposición cerca de la MAPE.

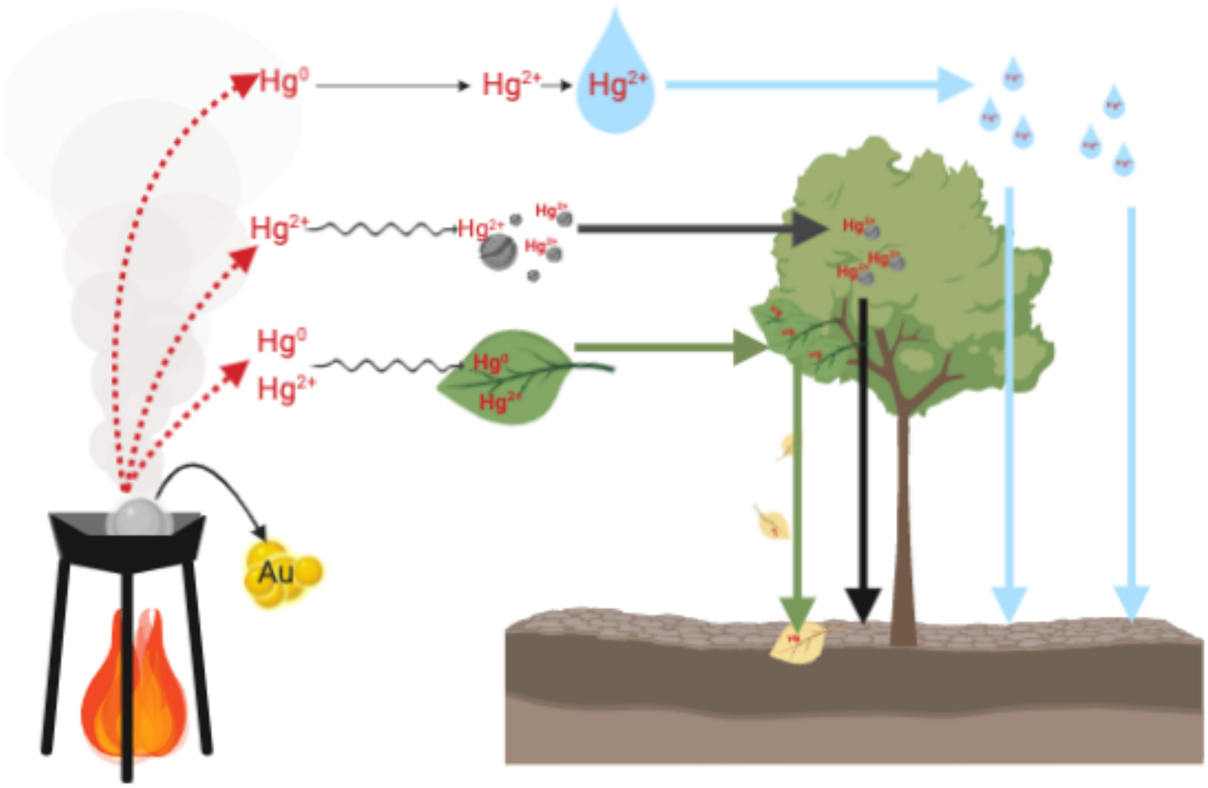

Figura 1: Formas de deposición del mercurio en el medio ambiente. El mercurio emitido como mercurio elemental gaseoso (MEG; Hg^0) puede tomar tres procesos atmosféricos para depositarse en la superficie. En primer lugar, el MEG se puede oxidar a Hg iónico (Hg^{2+}), ser arrastrado en las gotas de agua y removido como deposición húmeda o seca en las superficies foliares. En segundo lugar, el MEG puede absorber partículas atmosféricas (Hg_P) que son interceptadas por las hojas y, junto con el Hg iónico interceptado, se depositan en la superficie a través de la trascolación. En tercer lugar, el MEG puede ser absorbido en el tejido de la hoja y el Hg luego es depositado como hojarasca. La trascolación y la hojarasca juntas se consideran como una estimación de la deposición total de Hg. Aunque el MEG también puede difundirse y adsorberse directamente en el suelo y la hojarasca, [73] es probable que esta no sea una vía importante para la entrada de Hg en el ecosistema terrestre.

Es de esperarse que las concentraciones de Hg elemental gaseoso disminuyan con la distancia a las fuentes de emisión de Hg. Dado que dos de las tres formas por las cuales el Hg se deposita en la superficie (trascolación y hojarasca) dependen de la interacción del Hg con las superficies de las plantas, también anticipamos que la tasa de deposición de Hg en los

ecosistemas y el riesgo que representa para los animales esté fuertemente influenciado por la estructura de la vegetación, como lo sugieren observaciones en los bosques templados y boreales en latitudes septentrionales. [19] Sin embargo, también reconocemos que las actividades de la MAPE ocurren con frecuencia en bosques tropicales, donde la estructura del dosel y la abundancia relativa del área foliar expuesta son muy diferentes. La importancia relativa de las formas de deposición del Hg en estos ecosistemas aún no se ha cuantificado lo suficiente, particularmente en bosques cercanos a fuentes de emisión de Hg con una intensidad que rara vez se observa en los bosques del norte. En este estudio, por tanto, nos preguntamos: (1) ¿Cómo varían las concentraciones de mercurio elemental gaseoso y las formas de deposición con la proximidad a la MAPE y al índice de área foliar del dosel regional? (2) ¿El almacenamiento de Hg en el suelo está relacionado con los aportes atmosféricos? y (3) ¿Existe evidencia de que la bioacumulación de Hg sea elevada en los pájaros cantores que viven en los bosques cercanos a la actividad de la MAPE? Este estudio es el primero en examinar los aportes por deposición de Hg cerca de la actividad de la MAPE y cómo la cobertura del dosel se correlaciona con estos patrones, así como el primero en medir las concentraciones de metilmercurio (MeHg) en la superficie terrestre de la Amazonía peruana. Medimos el MEG en la atmósfera, junto con el total de Hg y MeHg en la precipitación, la trascolación, el follaje, la hojarasca y el suelo en hábitats boscosos y deforestados a lo largo de un segmento de 200 km del río Madre de Dios en el sureste del Perú. Planteamos la hipótesis de que la proximidad a la MAPE y los pueblos mineros donde se queman las amalgamas de Hg-Au serían los factores más importantes que impulsan las concentraciones atmosféricas de Hg (MEG) y la deposición húmeda de Hg (precipitación total). Debido a que la deposición seca de Hg (trascolación + hojarasca) está relacionada con la estructura del dosel, [17,20] también anticipamos que las áreas boscosas tendrían mayores aportes de

Hg que las áreas deforestadas cercanas, lo que sería particularmente preocupante dado el alto índice de área foliar y el potencial de captura de Hg en los bosques amazónicos prístinos. Además, inferimos que la fauna que vive en los bosques cerca de los pueblos mineros tendría un contenido de Hg más alto que la que vive en áreas alejadas de la minería.

Resultados y Discusión

Nuestra investigación se realizó en el sureste de la Amazonía peruana, en el departamento de Madre de Dios, donde se han deforestado más de 100,000 hectáreas por la MAPE aluvial [3] adyacente a, y a veces dentro de, tierras protegidas y reservas nacionales. La actividad de la MAPE a lo largo de los ríos en esta región de la Amazonía occidental ha aumentado drásticamente durante la última década [21] y se espera que continúe a medida que los precios del oro se mantienen altos y con un incremento de la conectividad a los centros urbanos a través de la Carretera Interoceánica. [3] Seleccionamos dos sitios sin explotación minera (Boca Manu y Chilive, aproximadamente a 100 km y 50 km de la MAPE, respectivamente), denominados en lo sucesivo como "sitios remotos", y tres sitios dentro de la zona minera o "sitios mineros" (Figura 2A). Dos de los sitios mineros están ubicados en bosques secundarios cerca de los pueblos de Boca Colorado y Laberinto, y uno de los sitios está localizado en un bosque prístino maduro de la Concesión para Conservación Los Amigos. Nótese que la emisión de vapor de Hg de la quema de amalgama de Hg-Au ocurre regularmente dentro de esta zona minera, así como en los sitios de Boca Colorado y Laberinto, aunque se desconocen las ubicaciones exactas y el número de ubicaciones ya que estas actividades son generalmente informales y clandestinas; por “actividad de la MAPE” nos referimos tanto a la minería como a la quema de amalgamas. En cada ubicación, instalamos muestreadores para deposición tanto en los claros (áreas deforestadas completamente desprovistas de plantas leñosas) como debajo del dosel de los árboles (áreas

boscosas) en la estaciones secas y lluviosas para un total de tres campañas de muestreo (cada una con una duración de 1 a 2 meses) para recolectar deposición húmeda y trascolación, respectivamente, y colocamos muestreadores pasivos de aire en los claros para recolectar MEG. En el segundo año, instalamos recolectores en seis parcelas boscosas adicionales en Los Amigos con base en las altas tasas de deposición medidas durante el primer año.

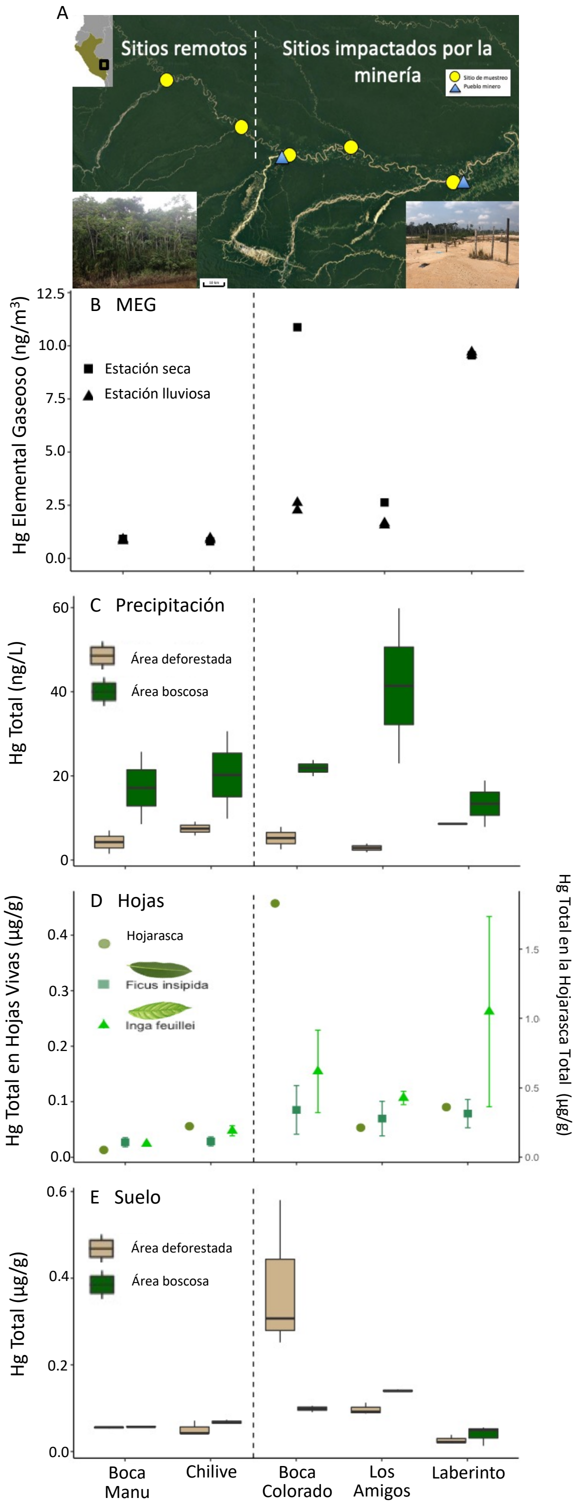

Figura 2: Concentraciones de mercurio en materiales depositados y suelo superficial en Madre de Dios, Perú. A) Mapa de los cinco sitios de muestreo. Dos sitios (Boca Manu, Chilive) están ubicados en áreas alejadas de la minería de oro artesanal y tres sitios (Los Amigos, Boca Colorado y Laberinto) están ubicados en áreas impactadas por la minería. Los recuadros muestran un sitio remoto boscoso típico y sitio deforestado impactado por la minería. En todas las figuras, la línea punteada representa la demarcación entre los dos sitios remotos (a la izquierda) y los tres

sitios impactados por la minería (a la derecha). B) Las concentraciones de mercurio elemental gaseoso (MEG) en cada sitio para las estaciones seca (n=1 por cada sitio) y lluviosa (n=2) de 2018. C) Concentración de mercurio total en precipitación recolectada en áreas boscosas y deforestadas durante la estación seca de 2018. Para todos los gráficos de caja, la línea representa el valor de la mediana, la caja muestra Q1 y Q3 y los bigotes denotan 1.5 veces el rango intercuartílico (n=5 para cada sitio boscoso, n=4 para cada sitio deforestado). D) Concentración de mercurio total en hojas recolectadas durante la estación seca de 2018 del dosel de *Ficus insipida* y *Inga feuillei* (eje izquierdo) y como hojarasca en el suelo (eje derecho). Los valores se muestran como promedio y desviación estándar (n=3 para hojas vivas en cada sitio, n=1 para hojarasca). E) Concentración de mercurio total en suelos superficiales (0-5 cm) recolectados durante la estación seca de 2018 (n=3 para cada sitio). Los datos para las otras estaciones se muestran en las figuras S1 y S2.

Las concentraciones de Hg atmosférico (MEG) corroboraron nuestras predicciones, con valores altos adyacentes a la actividad de la MAPE, particularmente cerca de los pueblos donde se queman las amalgamas de Hg-Au, y valores bajos en áreas alejadas de la minería activa (Figura 2B). En los sitios remotos, las concentraciones de MEG estaban por debajo del promedio de la concentración de base del hemisferio sur (~1 ng m^{-3}). [22] Por el contrario, las concentraciones de MEG en los tres sitios mineros fueron de 2 a 14 veces más altas que en los sitios remotos, con concentraciones en los sitios cercanos a los dos pueblos mineros (de hasta 10.9 ng m^{-3}) comparables, y en ocasiones superiores, a las concentraciones de áreas urbanas e industriales de los Estados Unidos, China y Corea del Sur. [23] Este patrón de MEG en Madre de Dios es consistente con la quema de amalgama de Hg-Au como la fuente principal del elevado Hg atmosférico en esta remota región amazónica.

El dosel del bosque como impulsor de la deposición de mercurio

Si bien las concentraciones de MEG en los claros se correlacionaron con su proximidad a la minería, las concentraciones de Hg total en la trascolación dependieron tanto de la proximidad a la minería como de la estructura del dosel del bosque. Este patrón sugiere que las concentraciones de MEG por sí solas no predicen en qué lugar de la región se depositará el Hg elevado. Medimos las concentraciones más altas de Hg en la trascolación en el bosque prístino maduro dentro de la zona minera (Figura 2C). La concentración promedio de Hg total en la trascolación en la Concesión para Conservación Los Amigos durante la estación seca estuvo

entre las más altas encontradas en la literatura (un rango de 18 a 61 ng L^{-1}), rivalizando con los niveles medidos en sitios contaminados por la minería de cinabrio y la combustión de carbón industrial en Guizhou, China, al considerar las diferencias en el volumen de precipitación. [24] Estos valores representan, hasta donde conocemos, el mayor flujo anual de Hg medido en la trascolación, basado en cálculos que utilizan las concentraciones de Hg y las tasas de precipitación de las estaciones secas y lluviosas (71 µg m^{-2} $año^{-1}$; Tabla S1). El Hg total en la trascolación en los otros dos sitios mineros no fue elevado en comparación con los sitios remotos (un rango de 8-31 ng L^{-1}; 22-34 µg m^{-2} $año^{-1}$). Aparte del Hg, solo los flujos del aluminio y manganeso fueron altos en la trascolación en los sitios mineros, lo que probablemente se deba al desmonte de tierras asociado con la minería; todos los demás elementos principales y traza medidos no variaron entre los sitios mineros y remotos (Data File S1), un hallazgo consistente con la dinámica del Hg foliar [25] y la quema de amalgama de la MAPE, en vez del polvo del aire como la principal fuente de Hg en la trascolación.

Las hojas de las plantas además de servir como absorbentes para partículas y Hg gaseoso, pueden asimilar MEG directamente e incorporarlo en los tejidos. [26,27] De hecho, la hojarasca fue una fuente importante de deposición de Hg en sitios cercanos a la actividad de la MAPE. Las concentraciones promedio de Hg medidas en las hojas vivas del dosel en los tres sitios mineros (0.080-0.22 µg g^{-1}) excedieron los valores publicados para bosques templados, boreales y alpinos en Norteamérica, Europa y Asia, así como otros bosques amazónicos en Suramérica, ubicados tanto en áreas remotas como en áreas con fuentes estacionarias cercanas. [28–30] Las concentraciones fueron comparables a las concentraciones de Hg foliar encontradas en bosques mixtos subtropicales en China y en el Bosque Atlántico de Brasil (Figura 2D). [28–30] Las concentraciones más altas de Hg total en la hojarasca total y las hojas del dosel se midieron en

los bosques secundarios dentro de la zona minera, siguiendo los patrones encontrados en MEG. Sin embargo, el flujo estimado de Hg en la hojarasca fue más alto en el bosque prístino maduro en la zona minera de Los Amigos, presumiblemente debido a una mayor masa de hojarasca. Estimamos que el flujo de Hg a través de la hojarasca en el sitio Los Amigos es de 66 µg Hg m^{-2} $año^{-1}$ tomando el Hg medido en la hojarasca (promediado entre las estaciones secas y lluviosas) y multiplicándolo por la masa de hojarasca previamente encontrada en la Amazonía peruana [31] (Figura 3A). Este aporte sugiere que tanto la proximidad a la minería como la cobertura del dosel son contribuyentes importantes a la carga de Hg de la MAPE en esta región.

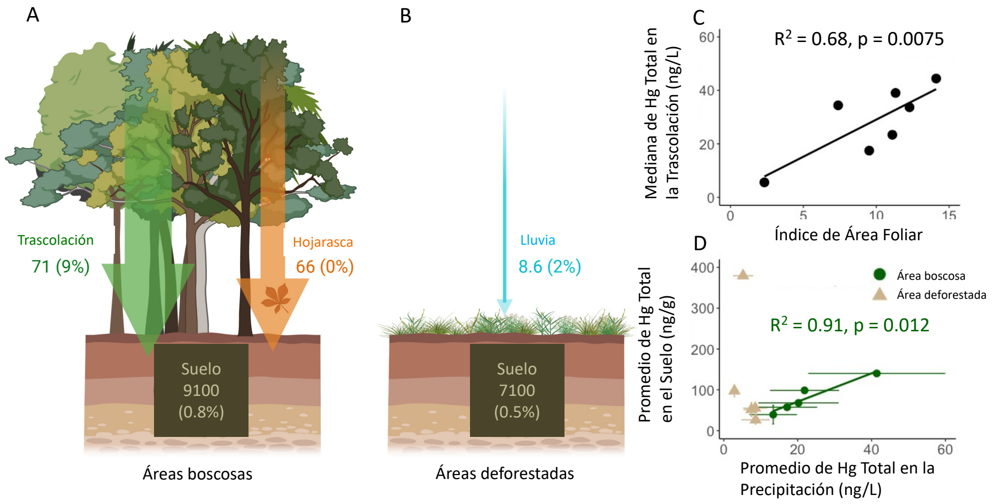

Figura 3: Flujo de mercurio y reservorio de mercurio en suelo superficial (0-5 cm) en la Concesión para Conservación Los Amigos en A) áreas boscosas y B) áreas deforestadas. El área deforestada en Los Amigos esta ubicada en un claro de la estación de campo que constituye una pequeña fracción de la superficie total. C) Relación entre la concentración de mercurio total en la trascolación y el índice de área foliar en las ocho parcelas de la Concesión para Conservación Los Amigos. D) Relación entre la concentración de mercurio total en la precipitación y la concentración de mercurio total en el suelo superficial en los cinco sitios en las áreas boscosas y deforestadas (las barras de error muestran la desviación estándar). Los flujos se muestran en flechas y se expresan como µg m^{-2} $año^{-1}$. Los reservorios se muestran en cuadrados para los primeros 0-5 cm del suelo y se expresan como µg m^{-2}. Los porcentajes representan el porcentaje de mercurio presente como metilmercurio en el reservorio o el flujo. La concentración promedio entre la estación seca (2018 y 2019) y la estación lluviosa (2018) para el mercurio total en la trascolación, la precipitación total y la hojarasca se utilizó para este escalamiento de la estimación de la carga de mercurio. Los datos de metilmercurio se basen en la estación seca de 2018, el único año en que se midió. Para obtener información sobre los cálculos del reservorio y el flujo, véase la sección de métodos.

Usando datos de precipitación y hojarasca recolectados a largo plazo, pudimos escalar nuestras mediciones de contenido de Hg en la trascolación y hojarasca derivadas de tres

campañas de muestreo para realizar una estimación preliminar del flujo de Hg atmosférico total anual (trascolación + hojarasca + precipitación) en la Concesión para Conservación Los Amigos. Encontramos que los flujos de Hg atmosférico en áreas de conservación boscosas adyacentes a la actividad de la MAPE son más de 15 veces mayores que las áreas deforestadas circundantes (137 vs. 9 µg Hg m^{-2} $año^{-1}$; Figura 3 A, B). Esta estimación preliminar de la carga de Hg en Los Amigos excede los flujos de Hg previamente encontrados en los bosques de Norteamérica y Europa cerca de fuentes estacionarias de Hg (por ejemplo, combustión de carbón) y está a la par con los valores en la China industrial. [17,32] En conjunto, aproximadamente 94% del Hg total depositado en los bosques conservados de Los Amigos se produce a través de la deposición seca (trascolación + hojarasca – Hg en la precipitación), una contribución mucho mayor que la deposición seca de los otros ecosistemas boscosos a nivel mundial. Estos resultados destacan la elevada cantidad de Hg de la actividad de la MAPE que ingresa a los bosques a través de la deposición seca y la importancia del dosel del bosque en la remoción atmosférica de Hg derivado de la MAPE. Anticipamos que los patrones observados de alta carga de Hg en la deposición en áreas boscosas cerca de la actividad de la MAPE no son aislados en el Perú.

En cambio, las áreas deforestadas en la zona minera tuvieron una carga de Hg más baja, en gran parte a través de la precipitación total con pocos aportes de Hg en la trascolación y hojarasca. Las concentraciones de Hg total en la precipitación total dentro de los sitios mineros fueron comparables a los valores medidos en los sitios remotos (Figura 2C). Las concentraciones promedio de Hg total en la precipitación total de la estación seca (un rango de 1.5 a 9.1 ng L^{-1}) estuvieron por debajo de los valores previamente encontrados en las Montañas Adirondack de Nueva York, [33] y generalmente por debajo de los valores para áreas remotas en el Amazonas. [34] Por lo tanto, en contraste con los patrones de concentración de MEG, trascolación y hojarasca en

los sitios mineros, los aportes de Hg en la precipitación total son uniformemente más bajas dentro de las áreas deforestadas adyacentes (8.6-21.5 µg Hg m^{-2} $año^{-1}$) y no reflejan proximidad a la minería. Debido a que la MAPE requiere deforestación, [2,3] las áreas despejadas donde se concentra la actividad minera reciben aportes de Hg más bajos por deposición atmosférica que las áreas boscosas cercanas, aunque las emisiones directas no atmosféricas de la MAPE, como el derrame de Hg elemental o los relaves, pueden ser altos. [18]

La variación en el flujo de Hg observada en la Amazonía peruana fue causada por grandes diferencias dentro y entre sitios (boscosos y deforestados) durante la estación seca (Figura 2). Por el contrario, vimos diferencias mínimas dentro y entre sitios y flujos bajos de Hg durante la estación lluviosa (Figura S1). Esta diferencia estacional (Figura 2B) probablemente se deba a una mayor intensidad tanto de la minería como de la generación de polvo durante la estación seca. El aumento de la deforestación y el bajo volumen de precipitación durante la estación seca probablemente incrementen la generación de polvo, aumentando así la cantidad de partículas atmosféricas que absorben Hg. Esta producción de Hg y polvo en la estación seca posiblemente conduzca a patrones en el flujo de Hg dentro de las áreas deforestadas en comparación con las áreas boscosas en la Concesión para Conservación Los Amigos.

Dado que los aportes de Hg de la MAPE en la Amazonía peruana se depositan en gran medida en los ecosistemas terrestres a través de la interacción con el dosel del bosque, examinamos si una mayor densidad del dosel (es decir, el índice de área foliar) conduciría a mayores aportes de Hg. Dentro del bosque prístino en la Concesión para Conservación Los Amigos, recolectamos trascolación de siete parcelas boscosas con diferentes densidades del dosel. Encontramos que el índice de área foliar es un fuerte predictor de los aportes de Hg total a través de la trascolación, con concentraciones medias de Hg total en trascolación que aumentan

con el índice de área foliar (Figura 3C). Muchas otras variables también impactaron las entradas de Hg a través de la trascolación, incluidas la edad de la hoja, [30] la rugosidad de la hoja, la densidad estomática, la velocidad del viento, [35] la turbulencia, la temperatura y el periodo seco anterior.

Destino del mercurio en los ecosistemas terrestres

En concordancia con las más altas tasas de deposición de Hg, los suelos superficiales (0-5 cm) del sitio boscoso en Los Amigos tuvieron las concentraciones de Hg total más altas (140 ng g^{-1} en la estación seca de 2018; Figura 2E) de nuestros sitios de estudio. Además, las concentraciones de Hg se incrementaron en todo el perfil de profundidad del suelo medido (con un rango de 138-155 ng g^{-1} hasta 45 cm de profundidad; Figura S3). El único sitio que exhibió mayores concentraciones de Hg en el suelo superficial durante la estación seca de 2018 fue un sitio deforestado cerca de un pueblo minero (Boca Colorado). En este sitio, planteamos la hipótesis de que las concentraciones extremadamente altas pueden deberse a la contaminación local de Hg elemental durante el proceso de amalgamación, ya que las concentraciones no se elevaron con la profundidad (>5 cm). También es probable que la fracción del Hg atmosférico depositado que retorna como emisión de Hg a la atmósfera, sea considerablemente menor en las áreas boscosas debido a la cobertura del dosel en comparación con las áreas deforestadas, [36] lo que sugiere que una fracción considerable del Hg depositado en las áreas de conservación se retiene dentro del suelo. El reservorio de Hg total del suelo en los bosques prístinos en la Concesión para Conservación Los Amigos fue de 9100 μg Hg m^{-2} de 0 a 5 cm y más de 80,000 μg Hg m^{-2} de 0 a 45 cm profundidad.

Dado que el follaje incorpora Hg predominantemente de la atmósfera y no del suelo [26,27] y luego entrega este Hg al suelo a través de la trascolación, es probable que las altas tasas de Hg

en la deposición estén impulsando los patrones observados en los suelos. Encontramos una fuerte correlación entre las concentraciones promedio de Hg total en suelo superficial y las concentraciones de Hg total en la trascolación en todos los sitios boscosos, y ninguna relación entre el Hg en el suelo superficial y las concentraciones de Hg total en la precipitación total en las áreas deforestadas (Figura 3D). También se encontraron patrones similares entre el reservorio de Hg del suelo superficial y el flujo de Hg total en la trascolación en áreas boscosas, pero no en las deforestadas (reservorio de Hg del suelo superficial vs. el flujo de Hg total de la precipitación total).

Casi todas las investigaciones sobre la contaminación terrestre por Hg asociada con la MAPE se han limitado a las mediciones de Hg total, aun cuando son las concentraciones de MeHg las que determinan la biodisponibilidad de Hg y la subsecuente exposición y acumulación trófica. En los ecosistemas terrestres, el Hg es metilado por microorganismos en condiciones anóxicas, [37,38] por lo que a menudo se asume que las concentraciones de MeHg son bajas en los suelos de las tierras altas. Sin embargo, documentamos por primera vez que existen concentraciones medibles de MeHg dentro de los suelos amazónicos cerca de la MAPE, lo que sugiere que las concentraciones elevadas de MeHg se extienden más allá de los ecosistemas acuáticos y tienen presencia en los ambientes terrestres dentro de estas áreas impactadas por la MAPE, incluidos los suelos que se inundan durante la estación lluviosa, así como aquellos que permanecen secos a lo largo del año. Las concentraciones más altas de MeHg en el suelo superficial durante la estación seca de 2018 se produjeron en dos de los sitios boscosos en la zona minera (Boca Colorado y la Concesión para Conservación Los Amigos; 1.4 ng MeHg g^{-1}, 1.4% Hg como MeHg y 1.1 ng MeHg g^{-1}, 0.79% Hg como MeHg). En vista de que estos porcentajes de Hg presentes como MeHg son comparables a otros sitios terrestres alrededor del

planeta (Figura S4), pareciera que las altas concentraciones de MeHg se deben a las altas entradas de Hg total y al alto almacenamiento de Hg total en el suelo, en lugar de una conversión neta eficiente de Hg inorgánico a MeHg (Figura S5). Nuestros resultados representan las primeras mediciones de MeHg en suelos cercanos a la MAPE en la Amazonía Peruana. Con base a otros estudios que encontraron una mayor producción de MeHg en áreas inundadas en comparación con secas [39,40], esperamos que las concentraciones de MeHg sean aún mayores en los humedales boscosos permanentes y estacionales cercanos que experimentan cargas similares de Hg. Aunque falta por determinar si el MeHg representa un riesgo de toxicidad para la vida silvestre terrestre cerca de la actividad minera de oro, estos bosques cercanos a la actividad de la MAPE podrían ser puntos críticos para la bioacumulación de Hg en las redes tróficas terrestres.

Implicaciones para los bosques tropicales y la biodiversidad

La implicación más importante y novedosa de nuestro trabajo es la documentación de cantidades elevadas de Hg depositadas en los bosques cercanos a la actividad de la MAPE. Nuestros datos muestran que este Hg está disponible y se mueve a través de las redes tróficas terrestres. Además, cantidades muy grandes de Hg se almacenan en la biomasa y los suelos con el potencial de ser emitidos con el cambio en el uso de la tierra [4] y los incendios forestales. [41,42] El sureste de la Amazonía peruana es uno de los ecosistemas más biodiversos del planeta en taxones de vertebrados e insectos. [43] La alta complejidad estructural dentro de los bosques tropicales prístinos promueve la biodiversidad de aves [44] y proporciona nichos para una amplia gama de especies que habitan en el bosque. [45] Por esta razón, más del 50% de la región de Madre de Dios está designada como tierra protegida o reserva nacional. [46] La presión internacional para controlar la actividad ilegal de la MAPE dentro de la zona de conservación amortiguadora de la Reserva Nacional de Tambopata ha crecido significativamente en los últimos diez años, lo que

resultó en una importante acción de aplicación de la ley por parte del gobierno peruano en 2019 (la Operación Mercurio). No obstante, de acuerdo con nuestras estimaciones preliminares, nuestros resultados sugieren que la propia complejidad del bosque, que es la base de la biodiversidad amazónica, hace que esta región sea altamente vulnerable a una mayor carga y almacenamiento de Hg a partir de las emisiones de Hg relacionadas con la MAPE, lo que condujo a encontrar los más altos flujos de Hg medidos en la trascolación a nivel mundial y un flujo elevado de Hg en la hojarasca en bosques prístinos cerca de la MAPE. Si bien nuestra investigación se llevó a cabo en un bosque protegido, el patrón de elevados aportes y retención de Hg se aplicaría a cualquier bosque primario cerca de la actividad de la MAPE, incluidas las zonas amortiguadoras, lo que hace que estos resultados sean relevantes tanto para los bosques protegidos como para los no protegidos. El riesgo que representa el Hg de la MAPE para la región es, por lo tanto, una función no sólo de los aportes directos de Hg a través de emisiones atmosféricas, derrames y relaves, sino también del potencial del ecosistema para capturar, almacenar y transformar Hg en la forma más biodisponible de MeHg, lo que sugiere impactos diferenciados para el reservorio global de Hg y la vida silvestre terrestre dependiendo de la cubierta forestal cercana a la minería.

Al secuestrar el Hg atmosférico, los bosques prístinos cerca de la MAPE pueden reducir el riesgo de Hg en los ecosistemas acuáticos cercanos y en el reservorio global de Hg atmosférico. Si estos bosques se talan para la expansión de actividades mineras o agrícolas, el Hg almacenado podría movilizarse del ecosistema terrestre al acuático a través de incendios forestales, emisión y/o escorrentía. [41,42,47–49] En la Amazonía peruana, donde se utilizan anualmente ~180 toneladas de Hg en la MAPE [50] y aproximadamente un cuarto de este Hg se emite a la atmósfera, [51] se necesitarían 30 millones de hectáreas de tierra boscosa prístina para

capturar todo este Hg dadas las altas tasas observadas en la Concesión para Conservación Los Amigos. Esta es un área aproximadamente 7.5 veces mayor que la extensión total de tierras protegidas y reservas naturales en la región de Madre de Dios (~4 millones de ha), un departamento que tiene la mayor fracción de tierra en estatus de protección que cualquier otro departamento peruano, y gran parte de esta tierra boscosa prístina no se encuentra dentro del radio de deposición de Hg de la MAPE. Por consiguiente, el secuestro forestal de Hg en bosques prístinos no es suficiente para evitar que el Hg derivado de la MAPE entre en el reservorio de Hg atmosférico regional y global, lo que sugiere la importancia de reducir las emisiones de Hg de la MAPE. El destino de la gran cantidad de Hg que se almacena en los ecosistemas terrestres está muy influenciado por las políticas de conservación. Las decisiones futuras con relación a cómo se manejan los bosques prístinos, particularmente en áreas adyacentes a la actividad de la MAPE, tienen implicaciones en la movilización y biodisponibilidad de Hg ahora y en las próximas décadas.

Incluso si los bosques pudieran secuestrar todo el Hg liberado en los bosques tropicales, esto no es una panacea para reducir la contaminación por Hg porque las redes tróficas terrestres también pueden ser vulnerables a la exposición al Hg. Sabemos poco sobre la concentración de Hg en la biota de estos bosques prístinos, pero estas primeras mediciones de la deposición de Hg terrestre y el MeHg del suelo sugieren que una alta carga de Hg y un MeHg elevado en los suelos podrían aumentar el riesgo de exposición en los consumidores de alto nivel trófico que habitan esos bosques. Los datos de estudios previos sobre bioacumulación de Hg terrestre en bosques templados encontraron que las concentraciones de Hg en la sangre de las aves estaban correlacionadas con las concentraciones de Hg en la deposición y que los pájaros cantores que consumen alimentos de origen terrestre pueden exhibir concentraciones elevadas de Hg. [52,53] La

elevada exposición al Hg en los pájaros cantores conduce a una reducción de la efectividad y el éxito reproductivo, una disminución de la supervivencia de la descendencia, un desarrollo deficiente, un comportamiento alterado, estrés fisiológico y mortalidad. [54,55] Si este patrón se mantiene para la Amazonía peruana, el alto flujo de Hg que ocurre en los bosques prístinos podría conducir a altas concentraciones de Hg en aves y otra biota y a potenciar efectos adversos en estos ecosistemas. Esta es una preocupación particular ya que esta es una región megadiversa. [56] Estos resultados resaltan la importancia de prevenir que la actividad de la MAPE ocurra dentro de las reservas nacionales y las zonas amortiguadoras que las rodean. La formalización de la actividad de la MAPE [15,16] podría ser un mecanismo para asegurar que las tierras protegidas no sean minadas.

Para evaluar si el Hg depositado en estas áreas boscosas está ingresando a las redes tróficas terrestres, medimos las concentraciones totales de Hg en las plumas de la cola de varias especies de pájaros cantores residentes de la Concesión para Conservación Los Amigos (impactada por la minería) y la Estación Biológica Cocha Cashu (bosque maduro no afectado), un sitio remoto río arriba ubicado140 km más allá de nuestro sitio de muestreo más alejado en Boca Manu. Para las tres especies, de las cuales se muestrearon múltiples individuos en cada sitio, las concentraciones de Hg fueron elevadas en las aves de Los Amigos en comparación con Cocha Cashu (Figura 4). Este patrón estuvo presente independientemente de los hábitos alimenticios, ya que nuestras muestras incluyeron invertívoros del sotobosque *Myrmotherula axillaris*, invertívoros seguidores de hormigas *Phlegopsis nigromaculata* y frugívoros *Pipra fasciicauda* (1.8 [n=10] vs. 0.9 $\mu g\ g^{-1}$ [n=2], 4.1 [n=10] vs. 1.4 $\mu g\ g^{-1}$ [n=2], y 0.3 [n=46] vs. 0.1 $\mu g\ g^{-1}$ [n=2], respectivamente). De diez individuos de *Phlegopsis nigromaculata* muestreados en Los Amigos, tres excedieron la CE10 (concentración efectiva en la cual el éxito reproductivo se

reduce en 10%), tres excedieron la CE20 y una excedió la CE30 (véanse los estándares de CE en Evers [54]), mientras que ninguno de los individuos de cualquiera de las especies en Cocha Cashu excedió la CE10. Estos hallazgos iniciales de concentraciones promedio de Hg 2 y 3 veces más altas en pájaros cantores de un bosque protegido adyacente a la actividad de la MAPE, con concentraciones individuales de hasta 12 veces más altas, plantean una preocupación considerable sobre el grado en el que la contaminación por Hg de la MAPE puede estar ingresando en las redes tróficas terrestres.

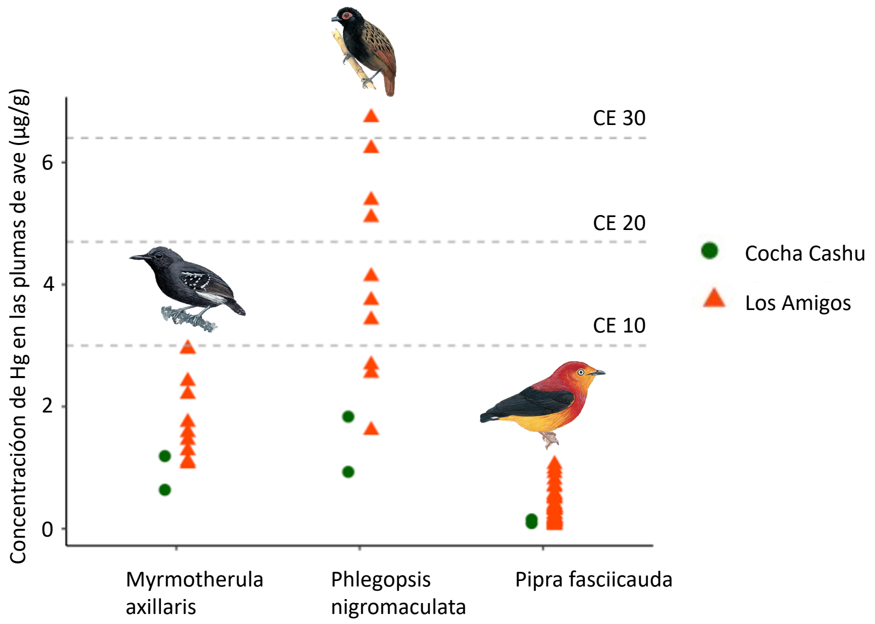

Figura 4: Concentraciones de mercurio total en plumas de la cola de especies de aves en la Concesión para Conservación Los Amigos (n=10 para *Myrmotherula axillaris* [invertívoro del sotobosque] y *Phlegopsi nigromaculata* [invertívoro seguidor de hormigas], n=46 para *Pipra fasciicauda* [frugívoro del sotobosque]) y el sitio remoto de la Estación Biológica Cocha Cashu (n=2 para cada especie). Se muestran las concentraciones efectivas (CE) en las que el éxito reproductivo se reduce en 10%, 20% y 30% (véase Evers [54]). Los fotos de las áves son modificadas de Schulenberg [61].

La extensión de la MAPE en la Amazonía peruana ha aumentado más de 40% en áreas protegidas desde 2012 y aún más en áreas no protegidas. [2,21] El uso continuo de Hg en la MAPE podría tener impactos devastadores en la vida silvestre que habita estos bosques. Incluso si los mineros eliminaran el uso de Hg de inmediato, este contaminante tiene una permanencia en los

suelos que puede extenderse por siglos, con potenciales pérdidas elevadas asociadas a la deforestación y los incendios forestales. [57,58] Por lo tanto, la contaminación por mercurio de la MAPE podría tener impactos duraderos en la biota de los bosques prístinos cercanos, tanto por los riesgos actuales como por el potencial de una contaminación futura a través de la liberación y la movilización de Hg, maximizados en bosques maduros con el mayor valor de conservación. Nuestro resultado de que la biota terrestre puede correr un riesgo considerable de contaminación por Hg derivado de la MAPE debería proporcionar un incentivo adicional a los esfuerzos en curso para reducir la emisión de Hg de la MAPE. Esos esfuerzos incluyen una variedad de enfoques que van desde el relativamente simple sistema de retortas para la captura de Hg hasta las más desafiantes inversiones sociales y económicas que formalizarían esta actividad y reducirían los incentivos financieros de llevar a cabo la MAPE ilegalmente.

Métodos

Recolección de muestras

Establecimos cinco sitios a lo largo de un tramo de 200-km del río Madre de Dios. Elegimos lugares de muestreo en función a su proximidad a actividades intensas de MAPE, con aproximadamente 50 km entre cada sitio de muestro y accesibles por el río Madre de Dios (Figura 2A). Seleccionamos dos sitios sin actividad minera (Boca Manu y Chilive, aproximadamente a 100 km y 50 km de la MAPE, respectivamente), a los que nos referiremos en adelante como “sitios remotos”. Seleccionamos tres sitios dentro de la zona minera, en lo sucesivo denominados como “sitios mineros”, con dos de los sitios ubicados en bosques secundarios cerca de los pueblos de Boca Colorado y Laberinto y uno ubicado en bosques intactos en la Concesión para Conservación Los Amigos. Nótese que la emisión de vapor de Hg de la quema de amalgama de Hg-Au ocurre regularmente dentro de esta zona minera, así como

en los sitios de Boca Colorado y Laberinto, aunque se desconocen las ubicaciones exactas y el número de ubicaciones ya que estas actividades son generalmente ilegales y clandestinas; por "actividad de la MAPE" nos referimos tanto a la minería como a la quema de amalgamas. En cada uno de los cinco sitios, instalamos muestreadores de deposición tanto en los claros (áreas deforestadas completamente desprovistas de plantas leñosas) como debajo del dosel del bosque (áreas boscosas) en la estación seca de 2018 (julio y agosto de 2018) y la estación lluviosa del 2018 (diciembre de 2018 y enero de 2019) para recolectar deposición húmeda (n=3) y trascolación (n=4), respectivamente. Se recolectaron muestras de precipitación en el transcurso de cuatro semanas en la estación seca y de dos a tres semanas en la estación lluviosa. En el segundo año de muestro durante la estación seca (julio y agosto de 2019), instalamos recolectores (n=4) en seis parcelas boscosas adicionales en Los Amigos por cinco semanas con base en las altas tasas de deposición medidas durante el primer año, para un total de siete parcelas boscosas y una parcela deforestada en Los Amigos. La distancia entre las parcelas osciló entre 0.1 y 2.5 km. Medimos las coordenadas en cada parcela utilizando un GPS Garmin portable.

Implementamos muestreadores pasivos de aire (MPA) para Hg en cada uno de los cinco sitios durante la estación seca de 2018 por un periodo de dos meses (de julio a agosto de 2018) y la estación lluviosa de 2018 por un mes (de diciembre de 2018 a enero de 2019). Se instaló un MPA por sitio durante la estación seca y varios MPA por duplicado durante la estación lluviosa. El MPA (desarrollado por McLagan et al. [59]) recolecta mercurio elemental gaseoso (MEG) por difusión pasiva a través de una barrera difusiva Radiello© y por absorción en un absorbente de carbono impregnado en azufre (HGR-AC). La barrera de difusión del MPA actúa como un obstáculo para evitar el paso de especies de Hg orgánico gaseoso; de modo que, solo el MEG se

absorbe en el carbono. [60] Adjuntamos MPA a postes aproximadamente a 1m por encima del suelo utilizando bridas de plástico. Todos los muestreadores se sellaron con *Parafilm* o se almacenaron en bolsas plásticas dobles y con cierre, antes y después del muestreo. Recolectamos blancos de MPA en campo y durante el viaje para evaluar la contaminación durante el almacenamiento de muestras en campo, en el laboratorio y durante el transporte de las muestras.

Durante los periodos de implementación en los cinco sitios de muestreo, colocamos tres recolectores de precipitación para el análisis de Hg y dos recolectores para otros análisis químicos en los sitios deforestados y cuatro recolectores de trascolación para análisis de Hg y dos recolectores para otros análisis químicos en los sitios boscosos. Los recolectores se colocaron a un metro de distancia entre sí. Nótese que, si bien instalamos una cantidad consistente de recolectores en cada sitio, durante algunos periodos de recolección tuvimos un tamaño de muestra más pequeño debido a la inundación de los sitios, la interferencia humana con los recolectores y el malfuncionamiento de la conexión entre el tubo y la botella recolectora. En cada sitio boscoso y deforestado, uno de los recolectores para el análisis de Hg contenía una botella de 500 mL, mientras que los otros contenían una botella de 250 mL; todos los recolectores para otros análisis químicos contenían una botella de 250 mL. Estas muestras se almacenaron frías hasta que el acceso a un congelador permitió congelarlas, transportarlas a los Estados Unidos en hielo y almacenarlas congeladas hasta su análisis. Los recolectores para el análisis de Hg consistieron en un embudo de vidrio conectado a una nueva botella de glicol de copoliéster de tereftalato de polietileno (PETG, por sus siglas en inglés) a través de tubo nuevo de polímero de bloque de estireno-etileno-butadieno-estireno (C-Flex ®) con un bucle como bloqueo de vapor. En el momento de la implementación, todas las botellas de PETG de 250 mL se acidificaron con 1 mL de ácido clorhídrico (HCI) con grado de metales traza y todas las

botellas de PETG de 500 mL se acidificaron con 2 mL de HCI con grado de metales traza. Los recolectores para otros análisis químicos consistieron en un embudo de plástico conectado a una botella de polietileno a través de un tubo nuevo C-Flex ® con un bucle como bloqueo de vapor. Antes de los muestreos, todos los embudos de vidrio, los embudos de plástico y las botellas de polietileno se lavaron con ácido. Recolectamos las muestras utilizando el protocolo manos limpias-manos sucias (Método EPA 1669), mantuvimos las muestras lo más frías posible hasta el regreso a los Estados Unidos, donde luego las almacenamos a 4°C hasta su análisis. Un estudio anterior que utilizó esta metodología ha demostrado que los blancos de laboratorio por debajo del límite de detección y los estándares añadidos tienen recuperaciones del 90-110%. [33]

En cada uno de los cinco sitios, recolectamos follaje como hojas del dosel, muestras de hojas individuales, hojarasca fresca y total utilizando el protocolo manos limpias-manos sucias (Método EPA 1669). Todas las muestras fueron recolectadas con un permiso de recolección del Servicio Nacional Forestal y de Fauna Silvestre (SERFOR) en el Perú y se importaron a los Estados Unidos con un permiso de importación del Departamento de Agricultura de ese país (USDA). Recolectamos hojas del dosel de dos especies de árboles que se encuentran en todos los sitios: una especie de árbol emergente (*Ficus insipida*) y un árbol de tamaño mediano (*Inga feuilleei*). Recolectamos hojas del dosel de los árboles (n=3 para cada especie) usando una honda Notch Big Shot ® en la estación seca de 2018, la estación lluviosa de 2018 y la estación seca de 2019. Recolectamos muestras de hojas individuales (n=1) mediante el muestreo de hojas en cada parcela de ramas de árboles a menos de dos metros del suelo en la estación seca de 2018, la estación lluviosa de 2018 y la estación seca de 2019. En 2019, también recolectamos muestras de hojas individuales (n=1) de las seis parcelas boscosas adicionales en Los Amigos. Recolectamos hojarasca (“hojarasca total”) fresca en cestas forradas con mallas de plástico (n=5) en la estación

lluviosa de 2018 en los cinco sitios boscosos y en la estación seca de 2019 en las parcelas de Los Amigos (n=5). Nótese que, si bien instalamos una cantidad consistente de cestas en cada sitio, durante algunos periodos de recolección tuvimos un tamaño de muestra más pequeño debido a la inundación de los sitios y la interferencia humana con los recolectores. Todas las cestas de hojarasca se colocaron a un metro de los recolectores de precipitación. Recolectamos hojarasca total como muestras al azar de hojarasca en el suelo en la estación seca de 2018, la estación lluviosa de 2018 y la estación seca de 2019. En la estación seca de 2019, también recolectamos hojarasca total en las parcelas de Los Amigos. Almacenamos en frío todas las muestras de hoja hasta que el acceso a un congelador permitió congelarlas, transportarlas a los Estados Unidos en hielo, para luego ser almacenadas congeladas hasta su procesamiento.

Recolectamos muestras de suelo por triplicado (n=3) en los cinco sitios (abiertos y en el dosel) durante las tres campañas estacionales y en las parcelas de Los Amigos en la estación seca de 2019. Todas las muestras de suelo se recolectaron a un metro de los recolectores de precipitación. Recolectamos muestras de suelo como suelo superficial debajo de la capa de hojarasca (0-5 cm) usando un barreno. Adicionalmente, en la estación seca de 2018, recolectamos núcleos de suelo de hasta 45 cm de profundidad y los dividimos en cinco segmentos de profundidad. En Laberinto, solo pudimos recolectar un perfil de suelo porque el nivel freático estaba cerca de la superficie del suelo. Recolectamos todas las muestras utilizando el protocolo manos limpias-manos sucias (Método EPA 1669). Almacenamos en frío todas las muestras de suelo hasta que el acceso a un congelador permitió congelarlas, transportarlas a los Estados Unidos en hielo, para luego ser almacenadas congeladas hasta su procesamiento.

Las aves fueron capturadas utilizando redes de niebla instaladas tanto al amanecer como al atardecer, durante las horas más frescas del día. En la Concesión para Conservación Los

Amigos, colocamos cinco redes de niebla (1.8 x 2.4) en nueve ubicaciones. En la Estación Biológica Cocha Cashu, colocamos de ocho a diez redes de niebla (12 x 3.2) en diecinueve ubicaciones. En ambos lugares, recolectamos la primera pluma central de la cola de cada ave, o si no estaba disponible, la siguiente pluma más antigua. Guardamos las plumas en bolsas Ziploc ® limpias o sobres manila con silicagel. Recopilamos un registro fotográfico y mediciones morfológicas para identificar la especie basándonos en Schulenberg [61]. Ambos estudios contaron con el respaldo de permisos del SERFOR y de los Comités Institucionales de Uso y Cuidado de Animales (IACUCs, por sus siglas en inglés). Al comparar las concentraciones de Hg de las plumas de aves, examinamos aquellas especies para las cuales se habían recolectado plumas tanto en la Concesión para Conservación Los Amigos como en la Estación Biológica Cocha Cashu (*Myrmotherula axillaris, Phlegopsis nigromaculata, Pipra fasciicauda)*.

Para determinar el Índice de Área Foliar (IAF), los datos lidar (del inglés "light detection and ranging") se recolectaron utilizando el laboratorio de vuelo no tripulado GatorEye, que es un sistema con sensores fusionados en un dron (detalles disponibles en www.gatoreye.org, con datos a escala de parcela disponibles para descarga usando el enlace "2019 Peru Los Amigos June) [62]. Los datos lidar se recolectaron en la Concesión para Conservación Los Amigos en junio de 2019 a 80 metros sobre el nivel del suelo, a una velocidad de vuelo de 12 m/s y con líneas de vuelo adyacentes a 100 m de distancia, lo que resultó en un porcentaje de cobertura del traslape lateral de 75%. La densidad de puntos superó los 200 puntos por m^2 distribuidos a lo largo del perfil vertical del bosque. El área de vuelo cubrió todas las parcelas de muestreo en Los Amigos para la estación seca de 2019.

Análisis de Laboratorio

Cuantificamos las concentraciones de Hg total de MEG recolectadas por MPA por desorción térmica, amalgamación y espectroscopía de absorción atómica (Método USEPA 7473) utilizando un instrumento Hydra C (Teledyne ®, CV-AAS). Realizamos la calibración de CV-AAS utilizando el Material de Referencia Estándar (en inglés SRM) 3133 del Instituto Nacional de Estándares y Tecnología de los Estados Unidos (NIST) (solución estándar de Hg, 10.004 mg g-1), con un límite de detección de 0.5 ng de Hg. Realizamos verificación de calibración continua (VCC) usando el NIST SRM 3133 y Estándar de Control de Calidad (en inglés QCS) usando NIST 1632e (carbón bituminoso 135.1 mg g^{-1}). Dividimos cada muestra en botes separados, la colocamos entre dos capas delgadas de polvo de carbonato de sodio (Na_2CO_3) y la cubrimos con una capa delgada de polvo de hidróxido de aluminio ($Al(OH)_3$). [63] Medimos todo el contenido de HGR-AC de cada muestra para eliminar cualquier falta de homogeneidad en la distribución de Hg dentro del absorbente del HGR-AC. Por lo tanto, calculamos la concentración de Hg para cada muestra con base en la suma del Hg total medido para cada bote y el contenido total del absorbente del HGR-AC en el MPA. Dado que para las mediciones de concentración solo se recolectó una muestra de MPA en la estación seca de 2018 para cada sitio, el control y aseguramiento de la calidad del método se llevó a cabo colocando las muestras entre los blancos realizados durante los procedimientos de muestro, los estándares internos y estándares de matriz coincidente. En la estación lluviosa de 2018, medimos muestras de MPA por duplicado. Los valores se consideraron aceptables cuando la diferencia porcentual relativa (DPR) medida tanto para la VCC como para estándares de matriz coincidente estaban dentro del 5% de los valores aceptados y todos los blanco realizados durante los procedimientos estaban por debajo del límite de detección (DLD). Corregimos el Hg total medido en MPA utilizando las concentraciones de los blancos de campo y de viaje (0.81 ± 0.18 ng g^{-1}, n=5). Calculamos las concentraciones de

MEG utilizando la masa total de Hg absorbido corregido dividido entre el tiempo de mustreo y la tasa de muestreo (volumen de aire despojado de Hg gaseoso por unidad de tiempo; 0.135 m^3 day^{-1}) [59,64] ajustada para la temperatura y el viento utilizando mediciones de temperatura y viento promedio para la región de Madre de Dios obtenidas de World Weather Online. El error estándar obtenido de las concentraciones de MEG medidas se basa en el error de los estándares externos medidos antes y después de las muestras.

Analizamos muestras de agua para determinar el Hg total mediante oxidación con cloruro de bromo por un mínimo de 24 horas, seguido de reducción y análisis de cloruro de estaño con purga y trampa, espectroscopía de fluorescencia atómica de vapor frío (en inglés CVAFS) y separación por cromatografía de gases (en inglés GC) (Método EPA 1631, revisión E) en un analizador automatizado de mercurio total Tekran ® 2600. Realizamos VCC para las muestras de la estación seca de 2018 utilizando el estándar certificado de Hg acuoso de Ultra Scientific® (10 µg L^{-1}) y verificación de calibración inicial (VCI) utilizando material de referencia certificado por el NIST 1641D (mercurio en agua, 1.557 mg kg^{-1}), con un límite de detección de 0.02 ng L^{-1}. Para las muestras de la estación lluviosa de 2018 y la estación seca de 2019, realizamos calibración y VCC utilizando el estándar de mercurio total de Brooks Rand Instrument ® (1.0 ng L^{-1}) y VCI utilizando el estándar de varios elementos en solución 2A por espectrometría de masas de plasma con acoplamiento inductivo (en inglés ICP-MS) de SPEX Centriprep ®, con un límite de detección 0.5 ng L^{-1}. Todos los estándares tuvieron recuperaciones dentro del 15% de los valores aceptados. Los blancos de campo, de digestión y de análisis estuvieron por DLD.

Liofilizamos las muestras de suelo y hojas durante cinco días. Homogeneizamos las muestras y luego las analizamos para determinar el Hg total en un analizador directo de mercurio

Milestone ® DMA-80 mediante descomposición térmica, reducción catalítica, amalgamación, desorción y espectroscopía de absorción atómica (Método EPA 7473). Para las muestras de la estación seca de 2018, realizamos calibración del DMA-80 utilizando NIST 1663c (cenizas volátiles de carbón, 1005 ng g^{-1}) y material de referencia certificado por el Consejo Nacional de Investigaciones de Canadá MEES-3 (sedimento marino, 91 ng g^{-1}). Realizamos VCC y MS utilizando NIST 1633c y QCS utilizando MEES-3, con un límite de detección de 0.2 ng Hg. Para las muestras de la estación lluviosa de 2018 y la estación seca de 2019, realizamos calibración del DMA-80 utilizando el estándar de mercurio total de Brooks Rand Instrument ® (1.0 ng L^{-1}). Realizamos VCC y MS utilizando NIST SRM 2709a (suelo de San Joaquin Soil, 1100 ng g^{-1}) y QCS utilizando DORM-4 (proteína de pescado, 410 ng g^{-1}), con un límite de detección de 0.5 ng Hg. Para todas las estaciones, analizamos las muestras por duplicado y aceptamos valores cuando la DPR entre dos muestras estaba dentro del 10%. Todos los estándares y adiciones en la matriz tuvieron recuperaciones dentro del 10% de los valores aceptados y todos los blancos estaban por DLD. Todas las concentraciones fueron expresadas en función a la masa seca.

Analizamos MeHg en muestras de agua y suelo colectadas durante las tres campañas de muestreo y en muestras de hoja durante la estación seca de 2018. Extrajimos muestras de agua con ácido sulfúrico con grado traza por un mínimo de 24 horas, [65] digerimos hojas con hidróxido de potasio al 2% en metanol a 55°C por un mínimo de 48 horas [66] y digerimos suelos mediante digestión por microondas con ácido HNO con grado de metal traza. [67,68] Analizamos las muestras de la estación seca de 2018 mediante acetilación acuosa con tetraetilborato de sodio, purga y trampa, y CVAFS en un espectrómetro Tekran ® 2500 (Método EPA 1630). Realizamos calibración y VCC utilizando estándares de MeHg de laboratorio certificados por Frontier Geosciences ® y QCS para sedimentos utilizando ERM CC580, con un límite de detección de

0.2 ng L^{-1}. Analizamos las muestras de la estación seca de 2019 mediante acetilación acuosa con tetraetilborato de sodio, purga y trampa, CVAFS, GC y ICP-MS en un Agilent ® 770 (Método EPA 1630). [69] Realizamos calibración y VCC utilizando el estándar de metilmercurio de Brooks Rand Instruments ®, con un límite de detección de 1pg. Para todas las estaciones, los estándares tuvieron una recuperación dentro del 15% de los valores aceptados y todos los blancos estuvieron por DLD.

Analizamos las plumas de ave para determinar el Hg total en analizador de mercurio directo Milestone ® DMA-80 mediante descomposición térmica, reducción catalítica, amalgamación, desorción y espectroscopía de absorción atómica (Método EPA 7473) en el Laboratorio de Toxicología del Instituto de Investigación de Biodiversidad (Portland, Maine, Estados Unidos), con un límite de detección del método de 0.001 $\mu g\ g^{-1}$. Realizamos VCC y QCS del DMA-80 con DOLT-5 (hígado de cazón, 0.44 $\mu g\ g^{-1}$) y CE-464. Todos los estándares tuvieron una recuperación dentro del 5% de los valores aceptados y todos los blancos estuvieron por DLD. Todos los duplicados estaban dentro de una DPR del 15%. Todas las concentraciones se expresaron como Hg total en plumas en función al peso fresco (pf).

Filtramos muestras de agua para otros análisis químicos con un filtro de membrana de 0.45 µm. Analizamos muestras de agua para determinar aniones (cloruro, nitrato, sulfato) y cationes (calcio, magnesio, potasio, sodio) mediante cromatografía iónica (Método EPA 4110B) [*USEPA*, 2017a] con un cromatógrafo de iones Dionex ® ICS 2000. Todos los estándares tuvieron recuperaciones dentro del 10% de los valores aceptados y todos los blancos estuvieron por DLD. Analizamos muestras de agua para determinar elementos de traza mediante espectrometría de masas de plasma acoplado inductivamente con un Thermofisher ® X-Series II.

Los estándares de calibración del instrumento se prepararon mediante dilución en serie del estándar de agua certificado NIST1643f. Todos los blancos estuvieron por DLD.

Análisis de datos y análisis estadísticos

Todos los valores de flujos y reservorios presentes en el texto y en las figuras se expresan en concentración promedio para las estaciones seca y lluviosa. Para estimaciones de los reservorios y flujos utilizando concentraciones mínimas y máximas medidas durante las estaciones secas y lluviosas (promediadas juntas para las dos estaciones para un flujo anual), véase la Tabla 1. Calculamos el flujo de Hg del bosque en la Concesión para Conservación Los Amigos como la suma de aportes de Hg a través de la trascolación y la hojarasca. Calculamos el flujo de Hg en áreas deforestadas a partir de la deposición de Hg en la precipitación total. Calculamos la precipitación promedio anual acumulada durante la última década (2009-2018) como aproximadamente 2500 mm $año^{-1}$ utilizando mediciones de lluvia diarias de Los Amigos (recolectada como parte de EBLA, disponibles en ACCA previa solicitud). Nótese que, en el año 2018, la lluvia anual estuvo cerca de este promedio (2468 mm), mientras que los meses más lluviosos (enero, febrero y diciembre) representaron aproximadamente la mitad de la precipitación anual (1288 mm del total de 2468 mm). Por consiguiente, utilizamos el promedio de las concentraciones de la estación seca y lluviosa para los cálculos de flujo y reservorio. Esto además nos permite no solo tener en cuenta las diferencias en la cantidad de precipitación entre las estaciones secas y lluviosas, sino también las diferencias en la extensión de la actividad de la MAPE entre estas dos estaciones. Dado que los valores encontrados en la literatura de estimaciones de flujos anuales de Hg en bosques tropicales varían con el escalamiento, por ejemplo usando concentraciones de Hg de la estación seca y lluviosa o solo de la estación seca; al comparar nuestros flujos de Hg con los valores en la literatura, sólo lo hacemos con aquellos

que recolectaron muestras tanto en la estación seca como en la lluviosa. Igualmente, reestimamos nuestro flujo utilizando solo las concentraciones de Hg de la estación seca cuando el otro estudio recolectó muestras solo en la estación seca (por ejemplo, [70]).

Para determinar la carga anual de Hg total en trascolación, precipitación total y hojarasca en Los Amigos, usamos la concentración de Hg total promedio entre la estación seca (promedio para 2018 y 2019 en todos los sitios de Los Amigos) y la estación lluviosa (promedio para 2018). Para la carga de Hg total en otros sitios, se utilizó la concentración promedio entre la estación seca de 2018 y la estación lluviosa de 2018. Para la carga de MeHg, utilizamos datos de la estación seca de 2018, el único año en el que se midió MeHg. Para estimar el flujo de Hg de hojarasca, utilizamos estimaciones de la tasa de hojarasca provenientes de la literatura de 417 g m^{-2} $año^{-1}$ en la Amazonía peruana y concentraciones de Hg recolectadas de las hojas en las cestas. [31] Para el reservorio de Hg del suelo en los 5cm superficiales del suelo, utilizamos Hg total medido en el suelo (estación seca de 2018 y 2019; estación lluviosa de 2018) y concentraciones de MeHg de la estación seca de 2018 con una densidad aparente estimada de 1.25 g cm^{-3} de la Amazonía brasileña. [71] Solo pudimos hacer estos cálculos del balance en nuestro sitio de estudio en Los Amigos donde se encuentran disponibles conjuntos de datos de lluvia a largo plazo y la estructura del bosque intacto permite el uso de estimaciones de hojarasca recolectadas previamente.

Procesamos las líneas de vuelo del lidar utilizando el algoritmo de flujo de trabajo de posprocesamiento multiescalar del GatorEye que calcula automáticamente nubes de puntos fusionados y productos ráster limpios, incluido un modelo de elevación digital (MED) con una resolución de 0.5x0.5 m. Utilizamos el MED y la nube de puntos lidar limpia (WGS-84, UTM 19S Metros) como aportes en el flujo de trabajo de Densidad del Área Foliar de GatorEye (en

inglés G-LAD) que calcula estimaciones de área foliar calibrada (m^2) por vóxel (m^3) desde la superficie hasta de la parte superior del dosel con una resolución de 1x1x1 metro, así como el IAF derivado (la suma del LAD dentro de cada columna vertical de 1x1 m). Luego, se extrajo el valor del IAF para el punto GPS de cada parcela.

Realizamos todos los análisis estadísticos utilizando el software estadístico R versión 3.6.1 [72] e hicimos todas las visualizaciones utilizando ggplot2. Realizamos pruebas estadísticas con un alfa de 0.05. Las relaciones entre dos variables cuantitativas se evaluaron mediante regresión de mínimos cuadrados ordinarios. Realizamos comparaciones entre sitios utilizando la prueba no paramétrica de Kruskal seguida de la prueba de Wilcox por pares.

Disponibilidad de los datos

Todos los datos incluidos en este manuscrito están disponibles en la sección de Información Complementaria. Los datos de precipitación están disponibles en Conservación Amazónica (ACCA) previa solicitud.

Materiales y Correspondencia: La correspondencia y las solicitudes de materiales deben ser enviadas a jgerson1@gmail.com.

Referencias

1. NRDC. *Artisanal Gold : Opportunities for responsible investment-Summary*. *Investing in Artisanal Gold Summary v8* (2016).

2. Asner, G. P. & Tupayachi, R. Accelerated losses of protected forests from gold mining in the Peruvian Amazon. *Environ. Res. Lett.* **12**, (2017).

3. Espejo, J. C. *et al.* Deforestation and forest degradation due to gold mining in the Peruvian Amazon : A 34-year perspective. *Remote Sens.* **10**, 1–17 (2018).

4. Gerson, J. R. *et al.* Artificial lake expansion amplifies mercury pollution from gold mining. *Sci. Adv.* **6**, eabd4953 (2020).

5. Dethier, E. N., Sartain, S. L. & Lutz, D. A. Heightened levels and seasonal inversion of riverine suspended sediment in a tropical biodiversity hot spot due to artisanal gold mining. *Proc. Natl. Acad. Sci. U. S. A.* **116**, 23936–23941 (2019).

6. Abe, C. A., Lobo, F. de L., Novo, E. M. L. de M., Costa, M. & Dibike, Y. Modeling the effects of land cover change on sediment concentrations in a gold-mined Amazonian basin. *Reg. Environ. Chang.* (2019).

7. UNEP. *Global mercury assessment*. (2018).

8. Markham, K. E. & Sangermano, F. Evaluating wildlife vulnerability to mercury pollution from artisanal and small-scale gold mining in Madre de Dios, Peru. *Trop. Conserv. Sci.* **11**, (2018).

9. Alvarez-Berríos, N. *et al.* Impacts of small-scale gold mining on birds and anurans near the Tambopata Natural Reserve, Peru, assessed using passive acoustic monitoring. *Trop. Conserv. Sci.* **9**, 832–851 (2016).

10. Ashe, K. Elevated mercury concentrations in humans of Madre de Dios, Peru. *PLoS One* **7**, 1–6 (2012).

11. Langeland, A., Hardin, R. & Neitzel, R. Mercury levels in human hair and farmed fish near artisanal and small-scale gold mining communities in the Madre de Dios River Basin, Peru. *Int. J. Environ. Res. Public Health* **14**, 302 (2017).

12. Gonzalez, D. J. X., Arain, A. & Fernandez, L. E. Mercury exposure, risk factors, and perceptions among women of childbearing age in an artisanal gold mining region of the Peruvian Amazon. *Environ. Res.* **179**, 108786 (2019).

13. Gutleb, A. C., Schenck, C. & Stalb, E. Giant otter (Pteronura brasiliensis) at risk? Total mercury and methylmercury levels in fish and otter scats, Peru. *Ambio* **26**, 511–514 (1997).

14. Júnior, J. A. M. A. Y., Quigley, H. & Hoogesteijn, R. Mercury content in the fur of jaguars (Panthera onca) from two areas under different levels of gold mining impact in the Brazilian Pantanal. *An. Acad. Bras. Cienc.* 1–11 (2017).

15. Lodenius, M. & Malm, O. Mercury in the Amazon. *Rev. Environ. Contam. Toxicol.* **157**, 25–52 (1998).

16. Driscoll, C. T., Mason, R. P., Chan, H. M., Jacob, D. J. & Pirrone, N. Mercury as a global pollutant: Sources, pathways, and effects. *Environ. Sci. Technol.* **47**, 4967–4983 (2013).

17. Paige Wright, L., Zhang, L. & Marsik, F. J. Overview of mercury dry deposition, litterfall, and throughfall studies. *Atmos. Chem. Phys.* **16**, 13399–13416 (2016).

18. Gerson, J. R., Driscoll, C. T., Hsu-kim, H. & Bernhardt, E. S. Senegalese artisanal gold mining leads to elevated total mercury and methylmercury concentrations in soils,

sediments, and rivers. *Elem. Sci. Anthr.* **6**, (2018).

19. Hsu-Kim, H. *et al.* Challenges and opportunities for managing aquatic mercury pollution in altered landscapes. *Ambio* **47**, 141–169 (2018).

20. Witt, E. L., Kolka, R. K., Nater, E. A. & Wickman, T. R. Influence of the forest canopy on total and methyl mercury deposition in the boreal forest. *Water. Air. Soil Pollut.* **199**, 3–11 (2009).

21. Asner, G. P., Llactayo, W., Tupayachi, R. & Luna, E. R. Elevated rates of gold mining in the Amazon revealed through high-resolution monitoring. *Proc. Natl. Acad. Sci. U. S. A.* **110**, 18454–18459 (2013).

22. Sprovieri, F. *et al.* Atmospheric mercury concentrations observed at ground-based monitoring sites globally distributed in the framework of the GMOS network. *Atmos. Chem. Phys.* **16**, 11915–11935 (2016).

23. Sprovieri, F., Pirrone, N., Ebinghaus, R., Kock, H. & Dommergue, A. A review of worldwide atmospheric mercury measurements. *Atmos. Chem. Phys.* **10**, 8245–8265 (2010).

24. Guo, Y. *et al.* Distribution and wet deposition fluxes of total and methyl mercury in Wujiang River Basin, Guizhou, China. *Atmos. Environ.* **42**, 7096–7103 (2008).

25. Jiskra, M. *et al.* A vegetation control on seasonal variations in global atmospheric mercury concentrations. *Nat. Geosci.* **11**, 244–250 (2018).

26. Fay, L. & Gustin, M. Assessing the influence of different atmospheric and soil mercury concentrations on foliar mercury concentrations in a controlled environment. *Water. Air. Soil Pollut.* **181**, 373–384 (2007).

27. Ericksen, J. A. *et al.* Accumulation of atmospheric mercury in forest foliage. *Atmos. Environ.* **37**, 1613–1622 (2003).

28. Fu, X. W. *et al.* Observations of atmospheric mercury in China: A critical review. *Atmos. Chem. Phys. Discuss.* **15**, 11925–11983 (2009).

29. Zhou, J. *et al.* Examination of total mercury inputs by precipitation and litterfall in a remote upland forest of Southwestern China. *Atmos. Environ.* **81**, 364–372 (2013).

30. Teixeira, D. C., Lacerda, L. D. & Silva-Filho, E. V. Mercury sequestration by rainforests: The influence of microclimate and different successional stages. *Chemosphere* **168**, 1186–1193 (2017).

31. Nebel, G., Dragsted, J. & Vega, A. S. Litter fall, biomass and net primary production in flood plain forests in the Peruvian Amazon. *For. Ecol. Manage.* **150**, 93–102 (2001).

32. Shanley, J. B. & Bishop, K. H. Mercury cycling in terrestrial watersheds. in *Mercury in the environment: Pattern and process* (ed. Bank, M.) 119–141 (University of California Press, 2012).

33. Gerson, J. R. *et al.* Deposition of mercury in forests across a montane elevation gradient: Elevational and seasonal patterns in methylmercury inputs and production. *JGR Biogeosciences* **122**, 1–18 (2017).

34. Fadini, P. & Jardim, W. Is the Negro River Basin (Amazon) impacted by naturally occurring mercury? *Sci. Total Environ.* **275**, 71–82 (2001).

35. Hartman, J. S. *et al.* Application of a rule-based model to estimate mercury exchange for three background biomes in the continental United States. *Environ. Sci. Technol.* **43**, 4989–4994 (2009).

36. Magarelli, G. & Fostier, H. Quantificacao de fluxos de mercurio gasoso na interface solo/atmosfera utilizando camara de fluxo dinamico: Aplicacao na bacia do Rio Negro. *Quim. Nova* **28**, 968–974 (2005).

37. Ullrich, S. M., Tanton, T. W. & Abdrashitova, S. A. Mercury in the aquatic environment: A review of factors affecting methylation. *Crit. Rev. Environ. Sci. Technol.* **31**, 241–293 (2001).

38. Hsu-Kim, H., Kucharzyk, K. H., Zhang, T. & Deshusses, M. A. Mechanisms regulating mercury bioavailability for methylating microorganisms in the aquatic environment: A critical review. *Environ. Sci. Technol.* **47**, 2441–2456 (2013).

39. Rudd, J. W. M. Sources of methyl mercury to freshwater ecosystems: A review. *Water, Air, Soil Pollut.* **80**, 697–713 (1995).

40. Roulet, M., Guimaraes, J. & Lucotte, M. Methylmercury production and accumulation in sediments and soils of an Amazonian floodplain - Effect of seasonal indundation. *Water Air Soil Pollut.* **128**, 41–60 (2001).

41. Biswas, A., Blum, J. D., Klaue, B. & Keeler, G. J. Release of mercury from Rocky Mountain forest fires. *Global Biogeochem. Cycles* **21**, 1–13 (2007).

42. Kumar, A., Wu, S., Huang, Y., Liao, H. & Kaplan, J. O. Mercury from wildfires: Global emission inventories and sensitivity to 2000–2050 global change. *Atmos. Environ.* **173**, 6–15 (2018).

43. Monzon, E. V. *Plan de negocios 2007-201: SIAMAZONIA*. (2007).

44. MacArthur, R. H. & MacArthur, J. W. On bird species diversity. *Ecology* **42**, 594–598 (1961).

45. Ozanne, C. H. P. *et al.* Biodiversity meets the atmosphere: A global view of forest canopies. *Science (80-.).* **301**, 183–186 (2003).

46. Vuohelainen, A. J., Coad, L., Marthews, T. R., Malhi, Y. & Killeen, T. J. The effectiveness of contrasting protected areas in preventing deforestation in Madre de Dios, Peru. *Environ. Manage.* **50**, 645–663 (2012).

47. Porvari, P., Verta, M., Munthe, J. & Haapanen, M. Forestry practices increase mercury and methyl mercury output from boreal forest catchments. *Environ. Sci. Technol.* **37**, 2389–2393 (2003).

48. Veiga, M. M. & Meech, J. A. Mercury pollution from deforestation. *Nature* **368**, 816–817 (1994).

49. Magarelli, G. & Fostier, A. H. Influence of deforestation on the mercury air/soil exchange in the Negro River Basin, Amazon. *Atmos. Environ.* **39**, 7518–7528 (2005).

50. Cardo, M. A. & Vargas, P. M. *Proyecto: Plan nacional de accion sobre mercurio en el sector de la mineria de oro artesanal y de pequena escala en el Peru*. (2017).

51. Veiga, M. M. & Chouinard, R. *Results of the awareness campaign and technology demonstration for artisanal gold miners: Summary report*. (2008).

52. Rimmer, C. C. *et al.* Mercury concentrations in Bicknell's thrush and other insectivorous passerines in montane forests of northeastern North America. *Ecotoxicology* **14**, 223–240 (2005).

53. Rimmer, C. C., Miller, E. K., McFarland, K. P., Taylor, R. J. & Faccio, S. D. Mercury bioaccumulation and trophic transfer in the terrestrial food web of a montane forest. *Ecotoxicology* **19**, 697–709 (2010).

54. Evers, D. *The effects of methylmercury on wildlife: A comprehensive review and approach for interpretation*. *Encyclopedia of the Anthropocene* **5**, (Elsevier Inc., 2018).

55. Ackerman, J. T. *et al.* Avian mercury exposure and toxicological risk across western North America: A synthesis. *Sci. Total Environ.* **568**, 749–769 (2016).

56. Myers, N., Mittermeier, R. A., Mittermeier, C. G., de Fonseca, G. A. B. & Kent, J. Biodiversity hotspots for conservation priorities. *Nature* **403**, 853–858 (2000).

57. Obrist, D. *et al.* A review of global environmental mercury processes in response to human and natural perturbations : Changes of emissions, climate, and land use. *Ambio* **47**, 116–140 (2018).

58. Saiki, M. K., Martin, B. A., May, T. W. & Alpers, C. N. Mercury concentrations in fish from a Sierra Nevada foothill reservoir located downstream from historic gold-mining operations. *Environ. Monit. Assess.* **163**, 313–326 (2010).

59. McLagan, D. S. *et al.* A high-precision passive air sampler for gaseous mercury. *Environ. Sci. Technol. Lett.* **3**, 24–29 (2016).

60. Stupple, G. W., McLagan, D. S. & Steffan, A. In situ reactive gaseous mercury uptake on radiello diffusive barrier, cation exchange membrane and teflon filter membranes during atmospheric mercury depletion events. in *14th International Conference on Mercury as a Global Pollutant* (2019).

61. Schulenberg, T. S. *et al. Birds of Peru*. (Princeton University Press, 2010).

62. Almeida, D. R. A. *et al.* Monitoring the structure of forest restoration plantations with a drone-lidar system. *Int. J. Appl. Earth Obs. Geoinf.* **79**, 192–198 (2019).

63. Biswas, A., Blum, J. D., Bergquist, B. A., Keeler, G. J. & Xie, Z. Natural mercury isotope

variation in coal deposits and organic soils. *Environ. Sci. Technol.* **42**, 8303–8309 (2008).

64. McLagan, D. S. *et al.* Global evaluation and calibration of a passive air sampler for gaseous mercury. *Atmos. Chem. Phys.* **18**, 5905–5919 (2018).

65. Munson, K. M., Babi, D. & Lamborg, C. H. Determination of monomethylmercury from seawater with ascorbic acid-assisted direct ethylation. *Limnol. Oceanogr. Methods* **12**, 1–9 (2014).

66. Hintelmann, H. & Nguyen, H. T. Extraction of methylmercury from tissue and plant samples by acid leaching. *Anal. Bioanal. Chem.* **381**, 360–365 (2005).

67. Tseng, C. M. *et al.* Rapid and quantitative microwaveassisted recovery of methylmercury from standard reference sediments. *J. Anal. At. Spectrom.* **12**, 629–635 (1997).

68. Rahman, M. & Kingston, H. Development of a microwave-assisted extraction method and isotopic validation of mercury species in soils and sediments. *J. Anal. At. Spectrom.* **20**, 183–191 (2005).

69. Hintelmann, H. & Evans, R. D. Application of stable isotopes in environmental tracer studies - Measurement of monomethylmercury (CH3Hg+) by isotope dilution ICP-MS and detection of species transformation. *Fresenius. J. Anal. Chem.* **358**, 378–385 (1997).

70. Fostier, A. H. *et al.* Mercury fluxes in a natural forested Amazonian catchment (Serra do Navio, Amapa State, Brazil). *Sci. Total Environ.* **260**, 201–211 (2000).

71. Batjes, N. H. & Dijkshoorn, J. A. Carbon and nitrogen stocks in the soils of the Amazon Region. *Geoderma* **89**, 273–286 (1999).

72. ‘R Core Team’. R: a language and environment for statistical computing. *R Foundation for Statistical Computing* (2014).

73. Obrist, D. *et al.* Mercury distribution across 14 U.S. Forests. Part I: Spatial patterns of concentrations in biomass, litter, and soils. *Environ. Sci. Technol.* **45**, 3974–3981 (2011).

Agradecimientos

Agradecemos a Ramiro Cordova Salas, Francisco Phuno Soncco, Bryan Huamantupa Rivera, Cecilio Huamantupa, Arianna Basto, Kelsey Lansdale, Melissa Marchese, Arabella Chen, Christian Lara, Annie Lee, Fernanda Machicao, Tatiana Manidis, Laura Naslund, al Centro de Innovación Científica Amazónica (CINCIA), a Conservación Amazónica (ACCA), a la Concesión para Conservación Los Amigos, y especialmente a nuestros técnicos peruanos por su asistencia en campo; a Brooke Hassett, Kim Hutchison, Gary Dwyer, Nelson Rivera, Faye Koenigsmark, Natalia Neal-Walthall, Austin Wadle, Rachel Coyte, Rand Alotaibi, y Arianna Agostini por su asistencia en el laboratorio; a Jennifer Swenson por sus consejos sobre sensores remotos; y a Marx Gómez-Liendo por traducir el manuscrito al español.

El financiamiento a JRG fue proporcionado por la Beca para Trabajo de Campo de Disertación del Instituto de Salud Global de Duke, el Programa de Doctorado del Instituto de Salud Global de Duke, Conexiones Bass de la Universidad de Duke, el Premio Tinker de Beca de Viaje de Investigación del Centro de Estudios Latinoamericanos y del Caribe de la Universidad de Duke, la Beca de capacitación e investigación del Centro de Estudios Internacionales y Globales de la Universidad de Duke, el Premio de viaje internacional de investigación de disertación de la Universidad de Duke, Becas de ayuda a la investigación de la Sociedad Geológica de América, el Fondo Lewis y Clark para la Exploración e Investigación de Campo, y la Beca de Investigación para estudiantes graduados de la Fundación Nacional de Ciencias (NSF). El

financiamiento a ESB fue proporcionado por una Beca del Fondo de Dotación de la Fundación en memoria de Josiah Charles Trent.

Contribuciones de los autores

JRG contribuyó con la concepción, el diseño del estudio, la recolección y análisis de los datos y la preparación del manuscrito. NS contribuyó con la concepción y la recolección y análisis de los datos. EUA contribuyó con el análisis de los datos. ENB, GE, GI, KL, MJM, AM y MW contribuyeron con la recolección y análisis de los datos. ESB contribuyó con la concepción y el diseño del estudio. AAZ, BB, CTD, DCE, LEF, HH, WP, MS, y CV proporcionaron apoyo logístico en el trabajo de campo y en los análisis de laboratorio. Todos los autores proporcionaron comentarios sobre el manuscrito.

Conflicto de Interés

Los autores declaran no tener conflictos de intereses.
