# Supplementary material for: Amazon forests capture high levels of atmospheric mercury pollution from artisanal gold mining
Source: Nat Commun. 2022 Jan 28;13:559. doi: 10.1038/s41467-022-27997-3 (PMC8799693; doi:10.1038/s41467-022-27997-3)
Supplement: Supplementary file 2 — Description of Additional Supplementary Files [file 41467_2022_27997_MOESM2_ESM.docx]

Supplementary Data File 1: Concentrations of analytes in throughfall and bulk precipitation collected from five sites across Madre de Dios during the 2018 dry season (July-August). Concentrations reported in this Supplementary Table are the average concentration for multiple collectors at each site deployed and collected on the same days. BDL signifies concentrations that are below detection limit.

Supplementary Data File 2: Total mercury concentration in throughfall and bulk precipitation collected from five sites across Madre de Dios during the 2018 dry season (July-August) and wet season (December-January).

Supplementary Data File 3: Total mercury concentration in throughfall and bulk precipitation collected from the Los Amigos plots during the 2019 dry season (July). BDL signifies concentrations that are below detection limit.

Supplementary Data File 4: Total mercury concentration in leaves (*Ficus insipida, Inga feuillei*) and litter collected from five sites across Madre de Dios during the dry season (July-August) and wet season (December-January).

Supplementary Data File 5: Concentration of analytes in soil collected from five sites across Madre de Dios during the dry season (July-August) and wet season (December-January). All reported concentrations are for dry soil mass. BDL signifies concentrations that are below detection limit.
